# USP5 Promotes Ripretinib Resistance in Gastrointestinal Stromal Tumors by MDH2 Deubiquition

**DOI:** 10.1002/advs.202401171

**Published:** 2024-07-08

**Authors:** Haoyu Sun, Zhiwei Cui, Chao Li, Zhishuang Gao, Jun Xu, Yibo Bian, Tianhao Gu, Jianan Zhang, Tengyun Li, Qianzheng Zhou, Dinghua Yang, Zhongyuan He, Bowen Li, Fengyuan Li, Zekuan Xu, Hao Xu

**Affiliations:** ^1^ Department of General Surgery The First Affiliated Hospital of Nanjing Medical University Nanjing 210029 China; ^2^ Jiangsu Key Lab of Cancer Biomarkers Prevention and Treatment Jiangsu Collaborative Innovation Center for Cancer Personalized Medical University Nanjing 211166 China; ^3^ Department of General Surgery Zhongshan Hospital Fudan University School of Medicine #180 Fenglin Road Shanghai 200032 China; ^4^ Department of Breast Surgery Key Laboratory of Breast Cancer in Shanghai Fudan University Shanghai Cancer Center Shanghai 200032 China; ^5^ Department of Oncology The Second Affiliated Hospital of Nanjing Medical University Nanjing 210029 China

**Keywords:** gastrointestinal stromal tumor, mesothelial and soft tissue tumors, ripretinib resistance, ubiquitination

## Abstract

Ripretinib, a broad‐spectrum inhibitor of the KIT and PDGFRA receptor tyrosine kinases, is designated as a fourth‐line treatment for gastrointestinal stromal tumor (GIST). It is tailored for patients resistant to imatinib, sunitinib, and regorafenib. As its increasing use, instances of resistance to ripretinib are becoming more frequent. Unfortunately, there are currently no scientifically mature treatment options available for patients resistant to ripretinib. Posttranslational modifications (PTMs) such as ubiquitination, in conjunction with its interplay with other modifications, play a collective role in regulating tumor initiation and progression. However, the specific association between ubiquitination and ripretinib resistance is not reported. Through proteome–ubiquitinome sequencing, increased levels of the USP5 protein and decreased ubiquitination in ripretinib‐resistant GISTs are detected. Subsequent examination of the mass spectrometry findings validated the interaction through which TRIM21 governs USP5 expression via ubiquitination, and USP5 regulates MDH2 expression through deubiquitination, consequently fostering ripretinib resistance in GIST. Moreover, ZDHHC18 can palmitoylate MDH2, preventing its ubiquitination and further increasing its protein stability. The research underscores the correlation between posttranslational modifications, specifically ubiquitination, and drug resistance, emphasizing the potential of targeting the USP5‐MDH2 axis to counteract ripretinib resistance in GIST.

## Graphical abstract

This study investigates the resistance mechanism of the fourth‐line drug ripretinib in gastrointestinal stromal tumors through proteome–ubiquitinome sequencing. The TRIM21‐USP5‐MDH2 axis is a key factor in regulating drug resistance is innovatively found. Moreover, it is found that ZDHHC18 can palmitoylate MDH2, synergizing with USP5's deubiquitinating activity. After that, accumulated MDH2 converts α‐KG into 2‐HG, thereby mediating ripretinib resistance in GIST.

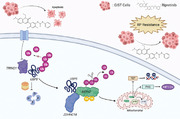

## 1 Introduction

Gastrointestinal stromal tumors (GISTs) are the predominant malignant mesenchymal tumors affecting the gastrointestinal tract, with an annual occurrence ranging from 6 to 22 cases per million individuals.^[^ ^1^ ^]^ ≈50–60% of gastrointestinal stromal tumors originate in the stomach, while 30–35% arise in the small intestine. Mutations in KIT are observed in 75–80% of patients, often impacting the juxtamembrane domain encoded by exon 11, while an additional 10% of patients exhibit PDGFRA mutations.^[^ ^2^ ^]^

Imatinib mesylate, a small‐molecule tyrosine kinase inhibitor (TKI), is utilized as the primary therapy for GIST since its ability for targen ted inhibition of KIT.^[^ ^3^ ^]^ IM improved the prognosis of patients significantly, with disease control observed in 70% to 85% of patients. Patients with metastatic GIST have a median progression‐free survival (PFS) time of 29 months and a median overall survival (OS) time of 57 months. However, complete tumor response (CR) is achieved in only 3–5% of patients receiving imatinib for advanced‐stage GIST, resulting in a 3‐year PFS rate of 31%.^[^ ^4^ ^]^ More than half of the patients who initially respond positively to imatinib treatment experience disease progression (PD) within a two‐year period.^[^ ^5^ ^]^ When patients experience disease progression or intolerance to imatinib, guidelines recommend sequential use of the second‐line drug sunitinib, third‐line drug regorafenib, and fourth‐line drug ripretinib (RP).^[^ ^6^ ^]^ For patients with advanced, imatinib‐refractory GIST, the clinical benefit rate was 53%, with a median progression‐free survival (PFS) time of 34 weeks upon receiving sunitinib as an alternative therapy. Regorafenib, a third‐line treatment, is used to treat patients with advanced GIST refractory to imatinib and sunitinib. The observed clinical benefit rate was 79%, with a median progression‐free survival (PFS) time of 10 months.^[^ ^7^ ^]^ Ripretinib is a potent inhibitor that targets the switch pocket control mechanism of the KIT and PDGFR kinases. It has been shown to demonstrate activity against a wide range of mutations, and is currently employed as a fourth‐line drug.^[^ ^8^ ^]^ For patients with GIST who progressed on or were intolerant to imatinib, sunitinib, and regorafenib, treatment with ripretinib significantly increased the PFS time to 6.3 months. Moreover, the median overall survival time was 15.1 months, and ripretinib had an objective response rate of 9%.^[^ ^9^ ^]^ However, for some patients whose condition progresses despite treatment with ripretinib, there is no precise precisely effective targeted drug available. Therefore, addressing ripretinib resistance and investigating the underlying mechanism are imperative.

Ubiquitin modification involves the interplay of E2 conjugating enzymes and E3 ligases, each of which possess distinct catalytic domains with unique enzymatic mechanisms, which are decoded by downstream machineries that selectively bind to these modifications and subsequently influence the fate of the modified proteins.^[^ ^10^ ^]^ Generally, the ubiquitin‒proteasome system is considered a double‐edged sword, that is capable of promoting tumor development in some instances while exerting inhibitory effects in others.^[^ ^11^ ^]^ It has been firmly linked to the onset and progression of various cancers, such as breast cancer, and pancreatic cancer.^[^ ^12^ ^]^ The sites where ubiquitin moieties attach to substrates determine the type of ubiquitination, including K48‐linked, K63‐linked, and K11‐linked ubiquitination. These various modes of ubiquitination govern specific functionalities and regulatory pathways.^[^ ^13^ ^]^ Indeed, the K48 linkage is the most prevalent type of linkage and is often associated with proteasomal degradation. This type of ubiquitination, i.e., K48‐linked ubiquitination, plays a critical role in numerous cancer‐related processes.^[^ ^14^ ^]^ Moreover, the ubiquitin‒proteasome pathway is increasingly being recognized for its role in overcoming resistance to targeted cancer therapies.^[^ ^15^ ^]^ Additionally, ubiquitination, a prominent posttranslational modification (PTM), can interact with other PTMs, resulting in alterations in the downstream target proteins.^[^ ^16^ ^]^

USP5 is a critical member of the USP subfamily, and plays a role in various tumors by targeting different proteins. For instance, it participates in USP5‐Beclin1 axis activation in non‐small cell lung cancer, reducing p53‐dependent senescence.^[^ ^17^ ^]^ Alternatively, it stabilizes SLUG to promote epithelial‐mesenchymal transition in hepatocellular carcinoma.^[^ ^18^ ^]^ However, USP5 has not been explicitly reported in GISTs.

In this study, we identified USP5 as a pivotal factor regulated by the upstream protein TRIM21, that mediates drug resistance by stabilizing the downstream target MDH2. Given that MDH2 undergoes palmitoylation mediated by ZDHHC18,^[^ ^19^ ^]^ we conducted further validation experiments to confirm the impact of palmitoylation on MDH2 ubiquitination and degradation.

## 2 Results

### 2.1 USP5 Promotes RP Resistance and Malignant Proliferation in GIST

**Figure 1:**
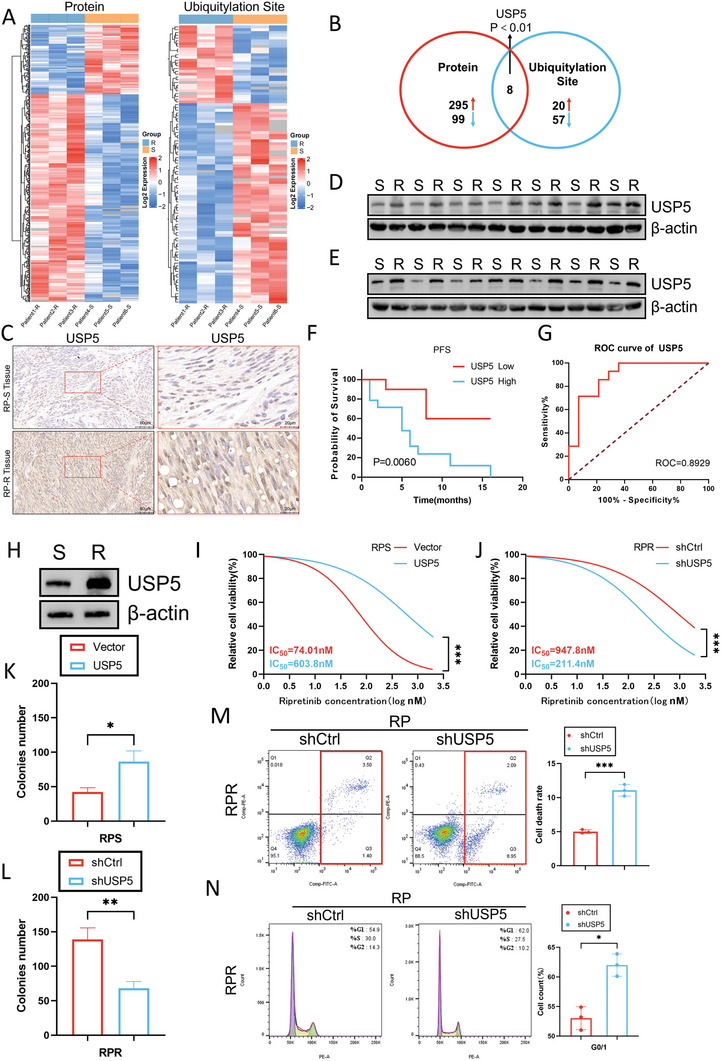
USP5 promotes RP resistance and the malignant proliferation of GIST. A) Heatmap representation of differentially expressed proteins and ubiquitin modification sites in patients with RP‐resistant and RP‐sensitive GIST. B) Venn diagram for differentially expressed proteins and ubiquitin modification sites in patients with RP‐resistant and RP‐sensitive GIST. C) IHC for USP5. D,E) Protein level of USP5 in human GIST tissues from RP‐resistant and RP‐sensitive patients. F) Kaplan–Meier plot of progression‐free survival by USP5 expression (Kaplan–Meier model with two‐sided log‐rank test). *p* = 0.0060. G) Receiver operating characteristic analysis of the risk of patients with RP‐sensitive and RP‐resistant GIST. H) Protein level of USP5 in GIST cells. I,J) CCK8 proliferation assay confirmed the influence of USP5 expression levels on the sensitivity of GIST cells to RP (mean ± SD, *n* = 3 independent experiments, two‐tailed Student's *t*‐test). ^***^ *p* < 0.001. K, L) Clone formation analysis verified the effect of USP5 expression levels on the proliferation of GIST cells after treatment with ripretinib (80 nm) for 24 h (mean ± SD, *n* = 3 independent experiments, two‐tailed Student's *t*‐test). ^*^ *p* < 0.05, ^**^ *p* < 0.01. M,N) Apoptosis rate and cell cycle distribution of RP‐resistant GIST cells transfected with shCtrl or shUSP5 after treatment with ripretinib (80 nM) for 24 h (mean ± SD, *n* = 3 independent experiments, two‐tailed Student's *t*‐test). ^*^ *p* < 0.05, ^***^ *p* < 0.001.

Through proteomic analysis comparing changes in protein expression and ubiquitination in 3 RP‐resistant and 3 RP‐sensitive GIST tissues, a total of 295 upregulated proteins, and 99 downregulated proteins, along with increased ubiquitination of 20 proteins and decreased ubiquitination of 57 proteins were identified in resistant tissues. Overlap analysis of the differentially expressed proteins and the proteins exhibiting altered ubiquitination revealed 8 distinct proteins, among which USP5 had the smallest *p*‐value, being the only protein with a *p*‐value less than 0.01. Thus, we selected USP5 as the target protein for further investigation (**Figure** **1**A,B). Next, immunohistochemical (IHC) staining revealed a direct relationship between the expression level of USP5 and heightened resistance to RP in GISTs (Figure 1C). Measurement of protein levels revealed a substantial increase in USP5 protein expression in RP‐resistant GIST tissues, but no alterations in its mRNA level (Figures 1D,E; Figure S1A, Supporting Information). This observation suggests that the increase in the USP5 protein level is not attributed to upregulation of USP5 mRNA transcription. Then, using the grayscale values of the proteins, the samples were divided into two distinct groups: a group with high‐expression of USP5 and a group with low‐expression of USP5. To assess the value of USP5 in predicting patient prognosis, we utilized ROC analysis. We found that USP5 could serve as a sensitive and promising biomarker for identifying RP‐resistant GISTs, as indicated by its area under the curve (AUC) of 0.8929 (95% CI, 0.7713 to 1.000) (Figure 1F,G). In general, the aforementioned findings suggest a potential correlation between increased expression of USP5 and resistance to RP in GIST.

To explore the resistance of GIST cells to ripretinib, we isolated primary human cells directly from tissues of ripretinib‐sensitive and ripretinib‐resistant stromal tumors and named them GIST‐RPS and GIST‐RPR, respectively. These primary stromal tumor cells exhibited a spindle‐shaped morphology when observed under a microscope (Figure S1B,C, Supporting Information). Immunostaining confirmed the positive expression of CD117 and DOG‐1, which are specific stromal tumor markers, but negative expression of markers of other cell types, such as immune cells, endothelial cells, and fibroblasts (Figure S1D, Supporting Information). Furthermore, genetic sequencing of these primary cell lines revealed no secondary mutations compared to the clinical samples (Table S1, Supporting Information). We separately examined primary cells, including ripretinib‐sensitive (RPS) and ripretinib‐resistant (RPR) GIST cells, and found that the protein expression of USP5 mirrored that observed in the tissues, with no difference in its mRNA level (Figure 1H; Figure S1E, Supporting Information). To investigate the correlation between USP5 expression and RP resistance in GIST, GIST‐RPS cells were transduced with the USP5 overexpression plasmid, while GIST‐RPR cells were transduced with short hairpin RNA (shRNA) targeting USP5. The efficiency of these transductions was demonstrated, and shUSP5‐1, which had the best transduction efficiency, was selected (Figure S1F–I, Supporting Information). Knockdown of USP5 notably reduced the half‐maximal inhibitory concentration (IC_50_) of RP. Conversely, overexpression of USP5 resulted in increased IC_50_ values in GIST‐RPS cells (Figure 1I,J). The results of the colony formation assay corroborated the aforementioned observations, underscoring the crucial involvement of USP5 in GIST proliferation (Figure 1K,L; Figure S1J,K, Supporting Information).

Flow cytometry was used to further investigate the effect of USP5 on proliferation, and the results indicated that overexpression of USP5 reduced the proportion of cells with G0/G1 arrest and significantly decreased the number of apoptotic cells. Conversely, transduction of shUSP5 diminished the effects of RP (Figure 1M,N; Figure S1L,M, Supporting Information). Furthermore, xenograft models were utilized to expand on the impact of USP5 in vivo. USP5 overexpression notably promoted tumor growth in nude mice, while transduction of shUSP5 decreased the growth rate of xenograft tumors (Figure S1N–P, Supporting Information). Overall, the presented data substantiate that USP5 promotes malignant proliferation and contributes to RP resistance in GIST cells.

### 2.2 TRIM21 Interacts with USP5 and Decreases its Stability

**Figure 2:**
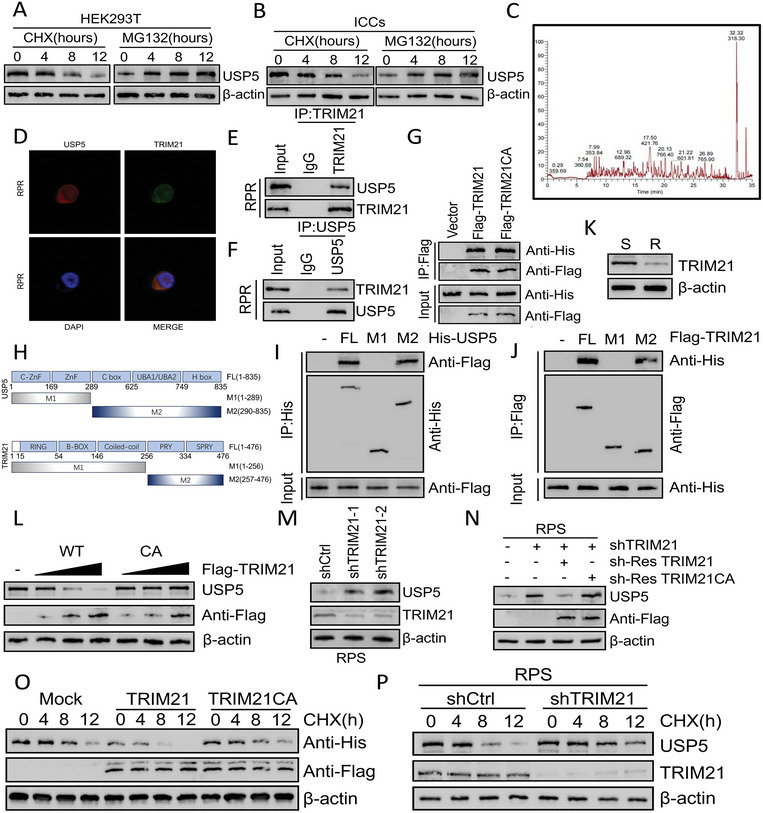
TRIM21 interacts with USP5 and maintains USP5 stability A, B Protein level of USP5 in HEK293T and ICCs cells after treatment with CHX (40 µg mL^−1^) or MG132 (20 µm). C) Base peak of USP5 binding proteins identified via a mass spectrometry assay. D) Confocal images showing colocalization of USP5 (red) and TRIM21 (green) in GIST‐RPR cells. E,F) Cell lysates from GIST‐RPR cells were analyzed by IP using antibodies against TRIM21 and USP5, then subjected to western blotting analysis. IgG was used as the isotype control. G HEK293T cells were transfected with His‐USP5 alone or in combination with Flag‐labeled wild‐type TRIM21 or TRIM21CA. Cell lysates were analyzed by IP with Flag beads followed by western blotting with antibodies against His and Flag. H) Schematic representation of full‐length (FL) Flag‐labeled TRIM21, His‐labeled USP5, and various deletion mutants. I) HEK293T cells were co‐transfected with His‐USP5 and FL Flag‐labeled TRIM21 or deletion mutants. Cell lysates were analyzed by IP with Flag beads followed by western blotting with antibodies against His and Flag. J) HEK293T cells were co‐transfected with Flag‐TRIM21 and His‐labeled FL USP5 or deletion mutants. Cell lysates were analyzed by IP with His beads followed by western blotting with antibodies against Flag and His. K) Protein level of TRIM21 in GIST cells. L) Increasing amounts of Flag‐labeled wild‐type TRIM21 or TRIM21CA were transfected into HEK293T cells, and cell lysates were analyzed by western blotting with antibody against USP5. M) GIST‐RPS cells were transfected with two independent TRIM21 shRNA and then TRIM21 and USP5 were analyzed. N) Protein level of USP5 in GIST‐RPS cells transfected with TRIM21 shRNA together with either shRNA‐resistant (sh‐res) Flag‐labeled wild‐type TRIM21 or TRIM21CA. O) HEK293T cells were co‐transfected with His‐labeled USP5 and Flag‐labeled wild‐type TRIM21 or TRIM21CA, treated with CHX (40 µg/ml), collected at the indicated times, and then subjected to western blotting with antibodies against His and Flag. P) GIST‐RPS cells stably expressing control shRNA or shRNA‐TRIM21 were treated with CHX (40 µg mL^−1^), harvested at the indicated times, and then subjected to western blotting with antibodies against USP5 and TRIM21.

To explore the intracellular regulation of USP5, we carried out the following experiments. We found that after inhibiting cellular protein synthesis using CHX, USP5 was gradually degraded over time. However, treatment with the proteasome inhibitor MG132 increased the USP5 protein level (**Figure** **2**A,B). According to the above observations, we inferred that the regulatory effect of USP5 occurred at the posttranslational level, likely through the ubiquitin‒proteasome system. Drawing upon the aforementioned findings, we identified proteins that interact with USP5 through mass spectrometry (MS) and immunoprecipitation (IP) analyses, which revealed the presence of the ubiquitination enzyme TRIM21 (Figure 2C; Table S2, Supporting Information). Hence, we speculated that TRIM21 plays a pivotal role in regulating USP5 stability. To investigate the interaction between TRIM21 and USP5, we analyzed their subcellular colocalization. Confocal microscopy revealed the coexistence of green fluorescence‐labeled TRIM21 and red fluorescence‐labeled USP5 in GIST cells within the cytoplasm (Figure 2D). Additionally, we verified the endogenous binding relationship between TRIM21 and USP5 proteins in GIST cells (Figure 2E,F; Figure S2A,B, Supporting Information). In vitro immunoprecipitation assays indicated that His‐labeled USP5 interacted with both wild‐type Flag‐labeled TRIM21 and TRIM21CA, suggesting that the binding of TRIM21 to USP5 is not associated with the ubiquitination activity of TRIM21 (Figure 2G). To delineate the interacting domains in TRIM21 and USP5, different deletions of Flag‐labeled TRIM21 and His‐USP5 were examined (Figure 2H). We observed that the amino‐terminal domain of USP5 (amino acids 290–835) and the M2 domain of TRIM21 (amino acids 257–476) primarily mediate the interaction between these two proteins (Figure 2I,J).

Continuing our investigation, we investigated whether TRIM21 has an influence on both the expression level and stability of USP5. Initially, we observed that TRIM21 expression was low in the RP‐resistant GIST cell line (Figure 2K). We showed that forced expression of wild‐type TRIM21 but not the catalytically inactive mutant TRIM21CA induced a dose‐dependent decrease in the endogenous USP5 protein level (Figure 2L). This finding suggests that TRIM21 regulates USP5 through its ubiquitination activity. TRIM21 depletion resulted in upregulation of USP5 expression, while expressing sh‐Res TRIM21 effectively reversed this effect; in contrast, the TRIM21CA mutant had no effect on USP5 expression (Figure 2M,N).

Moreover, CHX pulse‐chase experiments revealed that overexpressing wild‐type TRIM21 led to an accelerated degradation of USP5. In contrast, the overexpression of the TRIM21CA mutant did not affect the stability of USP5 (Figure 2O; Figure S2C, Supporting Information). Conversely, depletion of TRIM21 in sensitive cells enhanced the stability of the USP5 protein (Figure 2P; Figure S2D, Supporting Information). Taken together, these findings ultimately demonstrate that TRIM21 can decrease USP5 expression and play a regulatory role in controlling USP5 stability.

### 2.3 TRIM21 Regulates the Ubiquitination of USP5

**Figure 3:**
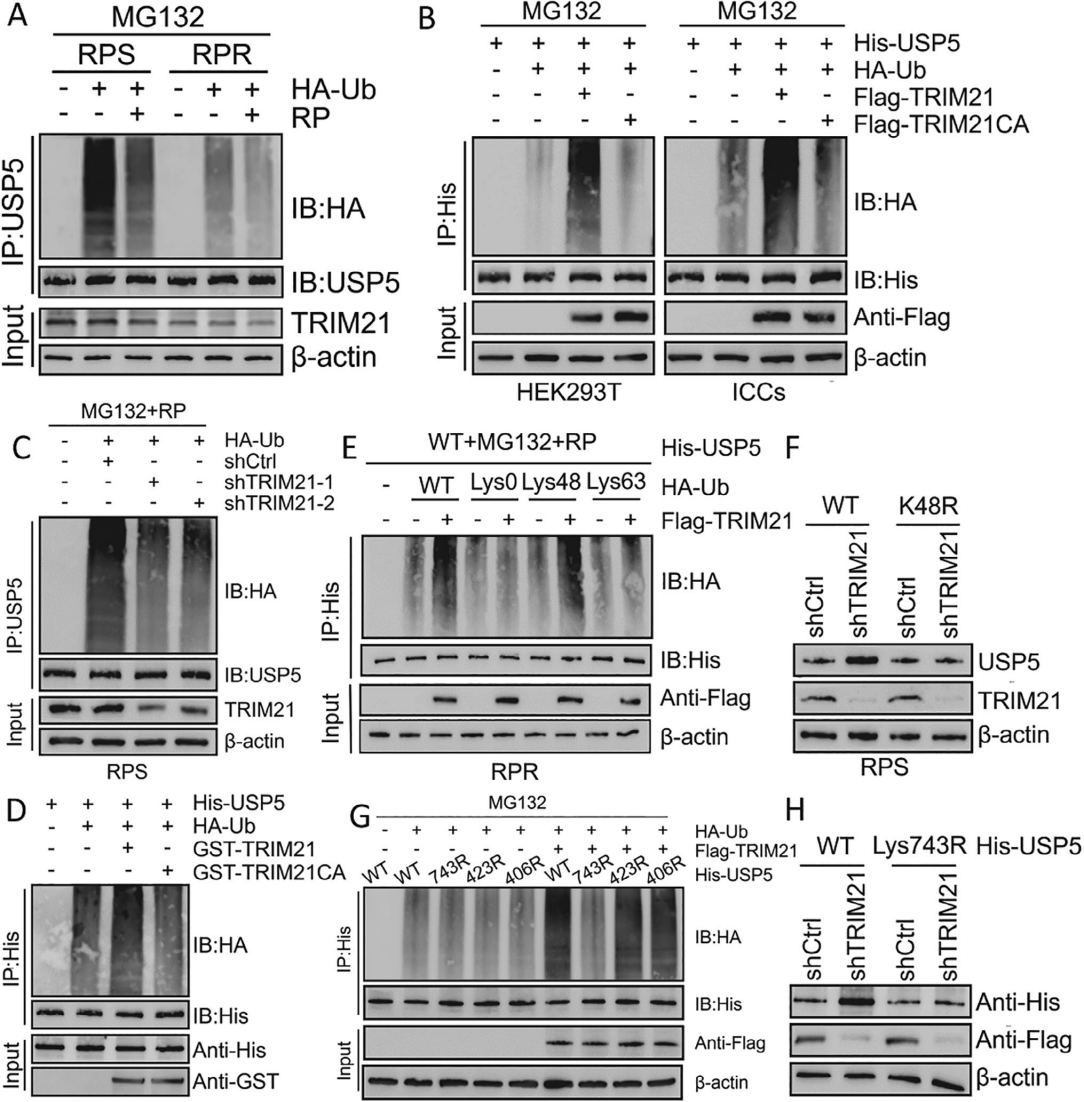
TRIM21 regulates the ubiquitination of USP5 A) GIST‐RPS and GIST‐RPR were co‐transfected with HA‐ubiquitin (HA‐Ub), and cell lysates were subjected to denature‐IP with USP5 antibody, followed by western blotting with antibodies against HA and USP5. Cells were treated with 20 µm MG132 and with or without low‐dose RP for 8 h before harvesting. B) HEK293T or ICCs cells were co‐transfected with His‐USP5, HA‐ubiquitin (HA‐Ub), and Flag‐labeled wild‐type TRIM21 or TRIM21CA, and cell lysates were subjected to denature‐IP with His beads followed by western blotting with antibodies against HA and His. Cells were treated with 20 µm MG132 for 8 h before harvesting. C) GIST‐RPS was co‐transfected with the indicated shRNA and HA‐Ub, and cell lysates were subjected to denature‐IP with USP5 antibody, followed by western blotting with antibodies against HA and USP5. Cells were treated with 20 µM MG132 for 8 h before harvesting. D) Unubiquitylated or ubiquitylated His‐USP5 was incubated with wild‐type GST‐TRIM21 or GST‐TRIM21CA coupled to glutathione‐sepharose beads. E) GIST‐RPR cells were co‐transfected with His‐USP5, Flag‐TRIM21, and HA‐Ub Lys0, Lys48‐only, or Lys63‐only plasmids, and then the USP5 ubiquitylation linkage was analyzed. F) GIST‐RPS cells transfected with wild‐type Ub or Ub‐Lys48R were cultured for 72 h in the presence of control shRNA or TRIM21 shRNA. Cell lysates were analyzed by western blotting using antibodies against USP5 and TRIM21. G) HEK293T cells were transfected with the vector plasmid or Flag‐TRIM21, HA‐Ub, and wild‐type His‐USP5 or K‐to‐R mutants and treated with a low‐dose of RP. Three lysine‐to‐arginine substitutions mutants of USP5 were established according to GPS‐Uber (http://gpsuber.biocuckoo.cn/wsresult.php). Samples were subjected to denature‐IP with anti‐Flag beads and then analyzed by immunoblot with an anti‐HA or anti‐Flag antibody. H) HEK293T transfected with wild‐type Ub, wild‐type His‐USP5 or His‐USP5‐K743R were cultured for 72 h in the presence of control shRNA or TRIM21 shRNA. Cell lysates were analyzed by western blotting using antibodies against USP5 and TRIM21.

The ubiquitination level of USP5 was lower in resistant cells than in sensitive cells. Moreover, treatment with RP alone also reduced the ubiquitination of USP5. Consistent with these findings, the protein level of TRIM21 was lower in the corresponding resistant cells, and treatment with RP alone also led to a decreased TRIM21 level (**Figure** **3**A). We conducted cotransduction experiments with wild‐type TRIM21 or the TRIM21CA mutant in both HEK293T and ICCs cells to verify the impact of these two proteins on the ubiquitination of USP5. IP assays revealed a notable increase in the ubiquitination of USP5 in cells cotransduced with wild‐type TRIM21 following treatment with MG132. In contrast, the ubiquitination level of USP5 in the TRIM21CA mutant group resembled that in the control group (Figure 3B). As expected, transduction of shTRIM21 in GIST‐RPS cells decreased the ubiquitination of USP5 (Figure 3C). To validate the direct association between USP5 and TRIM21, purified TRIM21 was incubated with ubiquitinated USP5 in a cell‐free system. The findings revealed that the increase in USP5 polyubiquitination in vitro occurred exclusively with purified wild‐type TRIM21, while the catalytically inactive mutant TRIM21CA did not produce the same effect (Figure 3D). Hence, these results indicate that TRIM21 directly ubiquitinates USP5.

Lys48 (K48) and Lys63 (K63) linkages are two well‐known primary polyubiquitin linkages.^[^ ^10^ ^]^ To determine the specific type of polyubiquitination through which TRIM21 impacts USP5 expression, we employed a set of ubiquitin mutants in which only one Lys residue was preserved and the rest of the lysine residues were substituted with Arg. TRIM21 facilitated the Lys48‐linked ubiquitination of USP5 but had no effect on its Lys63‐linked ubiquitination (Figure 3E). Moreover, overexpression of ubiquitin with Lys48 linkage‐resistant (K48R) ubiquitin but not of wild‐type (WT) ubiquitin inhibited the increase in USP5 expression induced by shTRIM21 (Figure 3F). Based on predictions from GPS‐Uber, we generated USP5 mutants with lysine‐to‐arginine substitution of the top three most likely E3‐specific lysine ubiquitination sites, and these three mutants, namely, USP5‐K743R, USP5‐K423R, and USP5‐K406R, were subjected to denaturing immunoprecipitation assays (Table S3, Supporting Information). Upon forced TRIM21 expression, the ubiquitination levels of wild‐type USP5 and USP5‐K423R/406R increased significantly, while the ubiquitination level of USP5‐K743R remained unchanged. Hence, TRIM21 specifically targets this mutant for ubiquitination (Figure 3G). Additionally, His‐USP5 expression was upregulated upon TRIM21 depletion, but this change was not observed in the presence of the K743R mutation (Figure 3H). In summary, this study reveals that TRIM21 promotes the degradation of USP5 via K48‐linked polyubiquitination at K743.

Furthermore, we overexpressed TRIM21 in RP‐resistant cells, as depicted in Figure S2E,F (Supporting Information). Our findings revealed that TRIM21 indeed decreased the IC_50_ of RP in RP‐resistant cells, resulting in reduced cell proliferation and increased sensitivity to ripretinib. However, notably, this effect was reversed by increasing the level of USP5 (Figure S2G–K, Supporting Information). These findings confirm that TRIM21 effectively modulates drug resistance in GIST cells by regulating the USP5 protein level.

### 2.4 USP5 Interacts with and Regulates the Stability of MDH2

**Figure 4:**
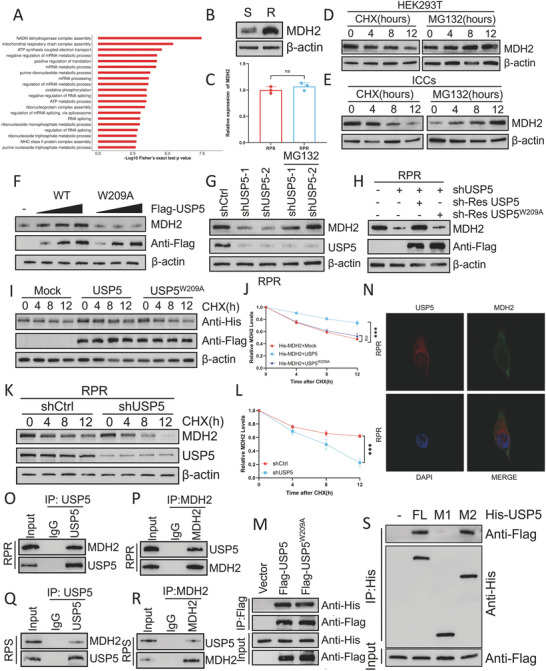
USP5 interacts with and regulates the stability of MDH2 A) Bar graph depicting the biological process of differentially expressed proteins in patients with RP‐resistant and RP‐sensitive GIST identified in GO enrichment analysis. B) Protein level of MDH2 in GIST cells. C) RT‐qPCR analysis of MDH2 target genes from GIST cells (mean ± SD, *n* = 3 independent experiments, two‐tailed Student's *t*‐test). ns: *p* ≥ 0.05. D,E) Protein level of MDH2 in HEK293T and ICCs cells after treatment with CHX (40 µg/ml) or MG132 (20 µm). F) Increasing amounts of Flag‐labeled wild‐type USP5 or USP5‐W209A were transfected into HEK293T cells, and cell lysates were analyzed by western blotting with an antibody against MDH2. G) GIST‐RPR cells transfected with two independent USP5 shRNA were treated with or without the proteasome inhibitor MG132 (20 µm for 8 h) and then USP5 and TRIM21 were analyzed. H) Protein level of MDH2 in GIST‐RPR cells transfected with USP5 shRNA together with either shRNA‐resistant (sh‐res) Flag‐labeled wild‐type USP5 or USP5‐W209A. I,J) HEK293T cells were co‐transfected with His‐labeled MDH2 and Flag‐labeled wild‐type USP5 or USP5‐W209A, treated with CHX (40 µg mL^−1^), collected at the indicated times, and then subjected to western blotting with antibodies against His and Flag. Quantification of MDH2 levels relative to β‐actin is shown (mean ± SD, *n* = 3 independent experiments, one‐way ANOVA with Dunnett's multiple‐comparison test). ^***^ *p* < 0.001 ns: *p* ≥ 0.05. K,L) GIST‐RPR cells stably expressing control shRNA or shRNA‐USP5 were treated with CHX (40 µg mL^−1^), harvested at the indicated times, and then subjected to western blotting with antibodies against MDH2 and USP5. Quantification of MDH2 levels relative to β‐actin is shown (mean ± SD, *n* = 3 independent experiments, two‐tailed Student's *t*‐test). ^***^ *p* < 0.001. M) HEK293T cells were transfected with His‐MDH2 alone or in combination with Flag‐labeled wild‐type USP5 or USP5‐W209A. Cell lysates were analyzed by IP with Flag beads followed by western blotting with antibodies against His and Flag. N) Confocal images showing colocalization of USP5 (red) and MDH2 (green) in GIST‐RPR cells. O–R) Cell lysates from GIST cells were analyzed by IP using antibodies against USP5 and MDH2, then subjected to western blotting analysis. IgG was used as the isotype control. S) HEK293T cells were co‐transfected with Flag‐MDH2 and His‐labeled FL USP5 or deletion mutants. Cell lysates were analyzed by IP with His beads followed by western blotting with antibodies against Flag and His.

To further investigate the mechanism through which USP5 regulates cellular resistance, we conducted functional enrichment analysis of the differentially expressed proteins between sensitive and resistant cells. By integrating the results with the mass spectrometry data for USP5, we identified MDH2 (Figures 2C and 4A). Afterward, we tested for heightened expression of MDH2 in RP‐resistant cells and detected no discernible difference in MDH2 mRNA expression (**Figure** **4**B,C). To prevent changes in de novo protein synthesis, we utilized CHX to track the MDH2 protein level in both HEK293T and ICCs cells. Upon CHX treatment, a progressive decrease in the MDH2 protein level was noted, and an extremely low level was attained within a span of 12 hs. Additionally, introduction of the proteasome inhibitor MG132 substantially increased the protein level of MDH2 (Figure 4D,E). These findings suggest that MDH2 may be posttranslationally regulated via the ubiquitin‒proteasome system. Consequently, we investigated the connection between MDH2 and USP5 by transducing HEK293T cells with Flag‐tagged USP5 or the catalytically dead USP5‐W209A mutant. Expression of wild‐type USP5 led to a marked dose‐dependent increase in MDH2 expression, whereas the W209A mutant did not exert this effect (Figure 4F). These findings suggested that USP5 regulates MDH2 through its deubiquitinase activity. Upon loss of USP5, there was a simultaneous decrease in the MDH2 protein level. However, this reduction was counteracted by MG132 treatment or by the introduction of wild‐type USP5 but not the USP5‐W209A mutant (Figure 4G,H). Furthermore, CHX pulse‐chase experiments performed with HEK293T cells demonstrated that overexpression of USP5 but not the USP5‐W209A mutant notably slowed the degradation of MDH2 (Figure 4I,J). Conversely, knocking down USP5 in RP‐resistant cells decreased the stability of MDH2 (Figure 4K,L). These findings strongly support the idea that USP5 is involved in governing the stability of MDH2.

Considering the potential binding relationship between MDH2 and USP5 detected previously (Figure 2C), the following experiments were conducted to verify this hypothesis and investigate the impact of the deubiquitination activity of USP5 on this binding interaction. Flag‐labeled wild‐type USP5 was found to interact with His‐labeled MDH2, as did Flag‐tagged USP5‐W209A, indicating that the catalytic site of USP5 has no effect on the binding of these proteins (Figure 4M). The subcellular colocalization of USP5 with MDH2 was further observed via confocal microscopy. As shown in Figure 4N, there was overlap between the USP5 (red) and MDH2 (green) signals within the cytoplasm. Likewise, we verified the interaction between the endogenous USP5 and MDH2 proteins (Figure 4O–R). To pinpoint the specific USP5‐binding region on MDH2, different His‐labeled USP5 constructs were generated (Figure 2H). IP assays revealed that the interaction between USP5 and MDH2 is predominantly mediated by the M2 domain of USP5 and particularly involves amino acids 290 to 835 (Figure 4S).

### 2.5 USP5 Regulates the Deubiquitination of MDH2

**Figure 5:**
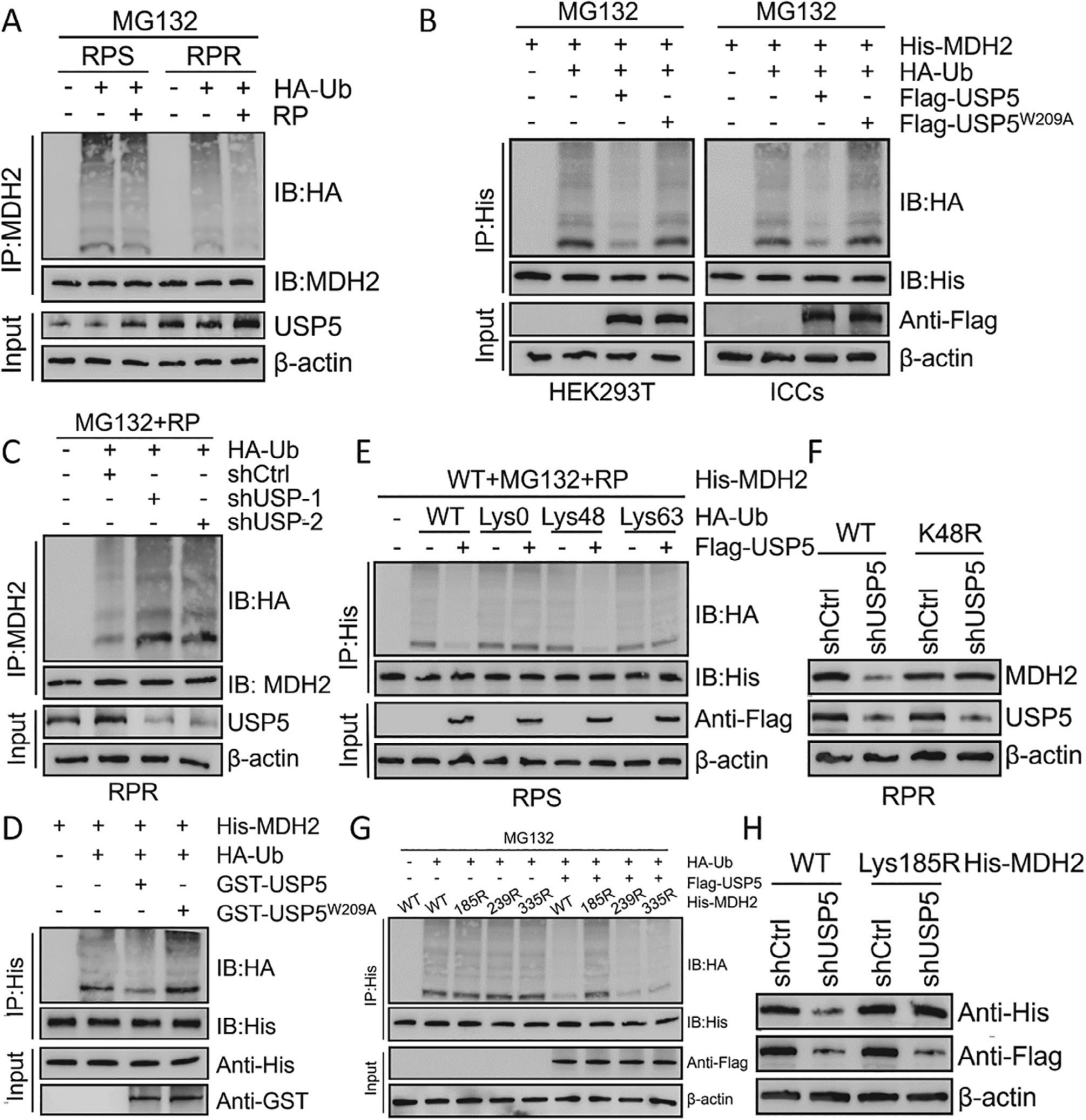
USP5 regulates the deubiquitination of MDH2 A) GIST‐RPS and GIST‐RPR were co‐transfected with HA‐ubiquitin (HA‐Ub), and cell lysates were subjected to denature‐IP with MDH2 antibody, followed by western blotting with antibodies against HA and MDH2. Cells were treated with 20 µm MG132 and with or without low‐dose RP for 8 h before harvesting. B) HEK293T or ICCs cells were co‐transfected with His‐MDH2, HA‐ubiquitin (HA‐Ub), and Flag‐labeled wild‐type USP5 or USP5‐W209A, and cell lysates were subjected to denature‐IP with His beads followed by western blotting with antibodies against HA and His. Cells were treated with 20 µm MG132 for 8 h before harvesting. C) GIST‐RPR was co‐transfected with the indicated shRNA and HA‐Ub, and cell lysates were subjected to denature‐IP with MDH2 antibody, followed by western blotting with antibodies against HA and MDH2. Cells were treated with 20 µm MG132 for 8 h before harvesting. D) Unubiquitylated or ubiquitylated His‐MDH2 was incubated with wild‐type GST‐USP5 or GST‐USP5‐W209A coupled to glutathione‐sepharose beads. E) GIST‐RPS cells were co‐transfected with His‐MDH2, Flag‐USP5, and HA‐Ub Lys0, Lys48‐only, or Lys63‐only plasmids, and then the MDH2 ubiquitylation linkage was analyzed. F) GIST‐RPR cells transfected with wild‐type Ub or Ub‐Lys48R were cultured for 72 h in the presence of control shRNA or USP5 shRNA. Cell lysates were analyzed by western blotting using antibodies against MDH2 and USP5. G) HEK293T cells were transfected with the vector plasmid or Flag‐USP5, HA‐Ub, and wild‐type His‐MDH2 or K‐to‐R mutants and treated with a low‐dose of RP. Three lysine‐to‐arginine substitution mutants of MDH2 were established according to GPS‐Uber (http://gpsuber.biocuckoo.cn/wsresult.php). Samples were subjected to denature‐IP with anti‐Flag beads and then analyzed by immunoblot with an anti‐HA or anti‐Flag antibody. H) HEK293T transfected with wild‐type Ub, wild‐type His‐MDH2 or His‐MDH2‐K185R were cultured for 72 h in the presence of control shRNA or USP5 shRNA. Cell lysates were analyzed by western blotting using antibodies against MDH2 and USP5.

Further investigations were conducted on the ubiquitination‐associated regulatory relationship between USP5 and MDH2. The results indicated that the MDH2 ubiquitination level was reduced in RP‐resistant GIST cells in comparison to RP‐sensitive GIST cells. Additionally, the ubiquitination level of MDH2 was observed to decrease upon treatment with RP. Similarly, RP‐resistant cells exhibited increased concentrations of USP5, and treatment with RP alone increased the level of USP5 (**Figure** **5**A). To assess whether USP5 is involved in the deubiquitination of MDH2, wild‐type USP5 or USP5‐W209A was transduced into HEK293T and ICCs cells. MDH2 cells subjected to MG132 treatment exhibited substantial ubiquitination, according to the IP assay. Cotransduction with wild‐type USP5 nearly completely halted the ubiquitination of MDH2, whereas cotransduction with USP5‐W209A did not have a similar effect (Figure 5B). Consistent with the experimental findings, shUSP5 increased the MDH2 ubiquitination level in RP‐resistant cells (Figure 5C). To ascertain whether MDH2 functions as a direct substrate of USP5, a cell‐free system was employed to incubate purified USP5 with ubiquitinated MDH2. In this experiment, purified wild‐type USP5 but not the catalytically inactive W209A mutant reduced MDH2 polyubiquitination in vitro (Figure 5D). Therefore, these findings confirm that USP5 directly participates in the deubiquitination of MDH2. Moreover, USP5 exclusively decreased the Lys48‐linked ubiquitination of MDH2 and did not impact its Lys63‐linked ubiquitination (Figure 5E). Furthermore, overexpression of the Lys48 linkage‐resistant (K48R) mutant of ubiquitin reversed the reduction in the MDH2 protein level triggered by shUSP5 (Figure 5F). Subsequently, we generated lysine‐to‐arginine substitution mutants of MDH2 based on predictions from GPS‐Uber (MDH2‐K185R, MDH2‐K239R, and MDH2‐K335R). These mutants were subjected to denaturing IP assays (Table S4, Supporting Information). In the absence of USP5 overexpression, all three mutants displayed ubiquitination, albeit to a lesser extent than did wild‐type MDH2. In the comparison of all experimental groups with USP5 overexpression, a decrease in ubiquitination was observed in each group except for the MDH2‐K185R‐ubiquitin group. This finding notably suggests that USP5 selectively targets this mutant for deubiquitination, which indicates that USP5 may exhibit higher deubiquitinase activity toward K185 (Figure 5G). Additionally, the reduction in His‐MDH2 expression due to USP5 depletion was subsequently reversed by the K185R mutation (Figure 5H). To summarize, our research findings provide evidence that USP5 increases the protein stability of MDH2 by cleaving K48‐linked polyubiquitin chains, specifically at the K185 residue.

### 2.6 MDH2 Induces RP Resistance in GIST

**Figure 6:**
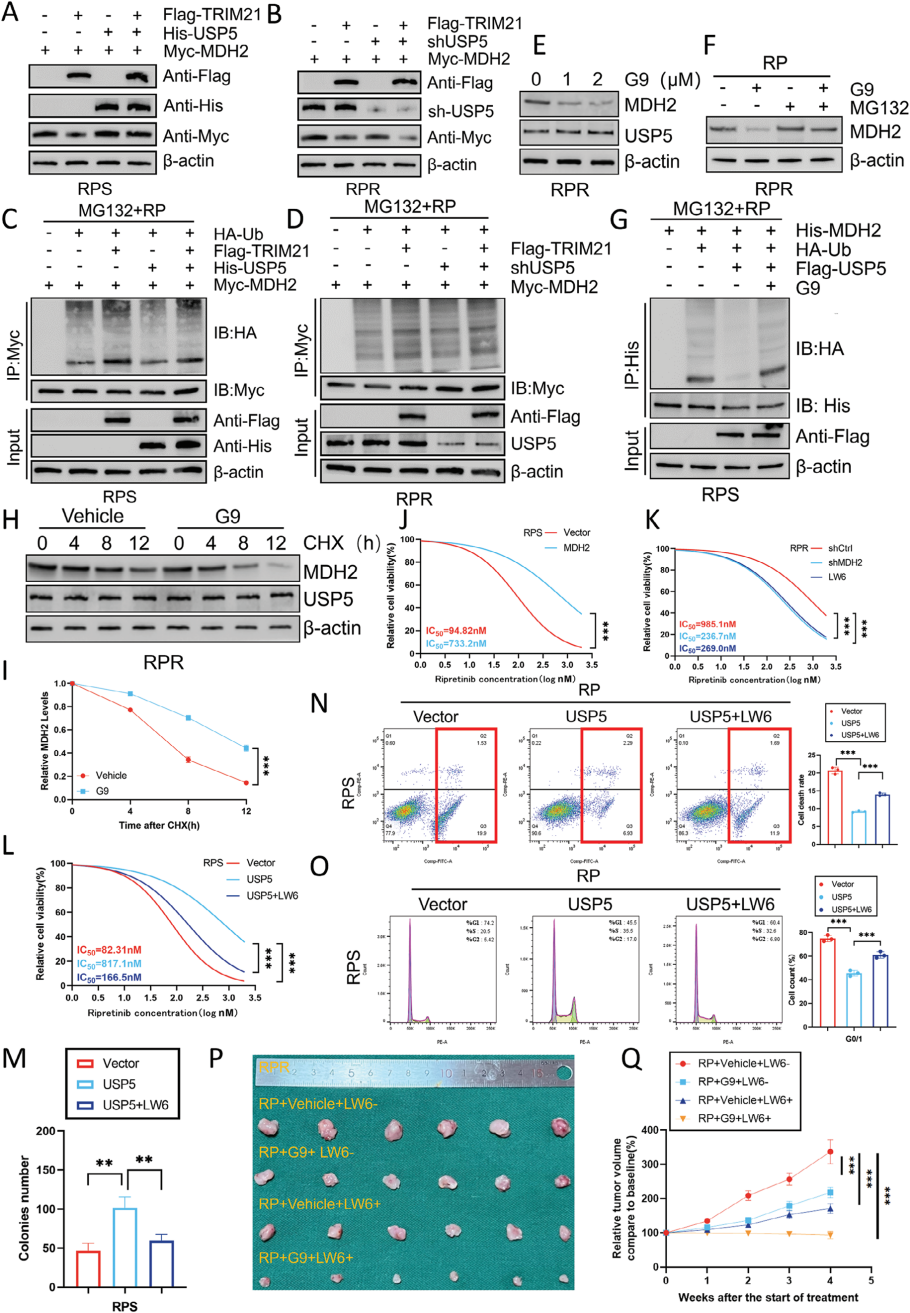
MDH2 induces RP resistance in GIST A,B) Western blotting assay was used to detect the effect of TRIM21 cooperating with USP5 on regulating MDH2 expression. C,D) GIST‐RPS and GIST‐RPR were co‐transfected with indicated plasmids. Cells were immunoprecipitated (IP) with an anti‐Myc antibody followed by immunoblotting against indicated antibodies after treatment with 20 µm MG132 and with low‐dose RP for 8 h. E) Western blot analysis of MDH2 and USP5 after treatment with different concentrations of G9. F) Western blot analysis of MDH2 in GIST‐RPR treated with G9 (2 µm) or vehicle with or without MG132. G) GIST‐RPS was co‐transfected with His‐MDH2, HA‐Ub, and Flag‐USP5 in the absence or presence of G9 (2 µm) or a low‐dose of RP and cell lysates were subjected to denature‐IP with His beads followed by western blotting with antibodies against HA and His. Cells were treated with 20 µm MG132 for 8 h before harvesting. H,I) GIST‐RPR cells were treated with G9 (2 µm) or vehicle for 24 h, followed by CHX (40 µg mL^−1^), harvested at the indicated times, and then subjected to western blotting with antibodies against MDH2. Quantification of MDH2 levels relative to β‐actin is shown (mean ± SD, *n* = 3 independent experiments, two‐tailed Student's *t*‐test). ^***^ *p* < 0.001. J) GIST‐RPS cells were transfected with a MDH2 overexpression vector or empty control. Cell viability of GIST cells after treatment is indicated (mean ± SD, *n* = 3 independent experiments, two‐tailed Student's *t*‐test). ^***^ *p* < 0.001. K) GIST‐RPR cells were transfected with shCtrl and MDH2 shRNA or treated with LW6 (30 µm). Cell viability of GIST cells after treatment is indicated (mean ± SD, *n* = 3 independent experiments, one‐way ANOVA with Dunnett's multiple‐comparison test). ^***^ *p* < 0.001. L) GIST‐RPS cells were transfected with a USP5 overexpression vector or empty control with or without LW6 (30 µm). Cell viability of GIST cells after treatment are indicated (mean ± SD, *n* = 3 independent experiments, one‐way ANOVA with Dunnett's multiple‐comparison test). ^***^ *p* < 0.001. M–O) Clone formation, apoptosis rate, and cell cycle distribution were assessed in GIST‐RPS cells transduced with empty vector or USP5 with or without LW6 (30 µm) after treatment with ripretinib (80 nm) for 24 h (mean ± SD, *n* = 3 independent experiments, one‐way ANOVA with Dunnett's multiple‐comparison test). ^**^ *p* < 0.01, ^***^ *p* < 0.001. P) Representative images of tumors in nude mice formed by the GIST‐RPR cells in the different subgroups. Q) Quantitative analysis of xenografted tumor growth rate and volume (mean ± SD, *n* = 6 mice for each group, one‐way ANOVA with Dunnett's multiple‐comparison test). ^***^ *p* < 0.001.

To investigate this phenomenon further, we analyzed the synergistic effect of TRIM21 and USP5 on MDH2 expression in GIST cells. Compared to the effects of individual overexpression of TRIM21 and deletion of USP5, significantly more pronounced increases in MDH2 degradation and ubiquitination were observed upon simultaneous overexpression of TRIM21 and deletion of USP5 (**Figure** **6**A,C). Additionally, transduction of shUSP5 mitigated the degradation and ubiquitination of MDH2 induced by overexpression of TRIM21 (Figure 6B,D).

To validate these previous findings and facilitate their clinical translation, we investigated the impact of pharmacological inhibition of USP5 on MDH2 expression in primary GIST cell lines. The small molecule USP5 inhibitor EOAI3402143 (G9), at a concentration of 2 µmol L^−1^, suppressed the expression of MDH2 and disrupted the deubiquitinase activity of USP5. As anticipated, G9 did not affect the expression level of USP5 (Figure 6E). G9 also decreased the expression of MDH2 in resistant GIST cells, and MG132 treatment reversed this reduction (Figure 6F). Like USP5 deletion, G9 facilitated the degradation of MDH2 through the ubiquitin‒proteasome pathway (Figure 6G). Furthermore, cotreatment with G9 and CHX notably reduced the protein half‐life of MDH2 (Figure 6H,I).

To explore the function of MDH2 in GIST, we established GIST‐RPS cells overexpressing MDH2 and employed the more efficient shMDH2‐2 to knock down MDH2 in resistant cells (Figure S3A–D, Supporting Information). Overexpression of MDH2 effectively increased the IC_50_ in RP‐sensitive cells, while knocking out MDH2 in resistant cells had the opposite effect (Figure 6J,K). Compared to control cells, GIST‐RPS‐MDH2 cells exhibited greater drug resistance, higher viability, and a greater proliferation ability. Conversely, knockdown of MDH2 in RP‐resistant cells had the opposite effects (Figure S3E–L, Supporting Information). Additionally, LW6, an MDH2 inhibitor,^[^ ^20^ ^]^ effectively reduced MDH2 activity at a concentration of 30 µm (Figure S3M, Supporting Information). The efficacy of LW6 was similar to that of shMDH2 in RP‐resistant cells (Figure 6K; Figure S3F,H,K,L, Supporting Information). Furthermore, we observed that LW6 reversed the increase in ripretinib resistance induced by USP5 and exerted a series of effects on increasing cell viability (Figure 6L–O, Figure S3N, Supporting Information). These results support our hypothesis that MDH2 plays a pivotal role in ripretinib resistance in GIST cells.

Based on the prior in vitro experimental results, we established an in vivo model of RP‐resistant subcutaneous tumors in nude mice for further in vivo investigations. Compared to the untreated RP‐resistant cell‐derived tumors, tumors treated with either LW6 or G9 exhibited increased sensitivity to RP and a reduced tumor propagating potential. Moreover, the xenografted nude mice showed smaller tumor volumes and prolonged survival when treated with both agents in combination (Figure 6P,Q).

Additionally, MDH2 has been reported to exhibit enzymatic promiscuity, allowing the conversion of α‐KG to 2‐HG.^[^ ^21^ ^]^ We also confirmed this phenomenon in stromal tumor cells by observing the decrease in α‐KG and increase in 2‐HG after knocking down USP5 or MDH2. In sensitive cells, knocking out MDH2 reversed the reduction in α‐KG and the increase in 2‐HG caused by overexpression of USP5 (Figure S3O–R, Supporting Information). Supplementation with DM‐αKG reduced the IC_50_ of RP in resistant stromal tumors, increasing their sensitivity to RP (Figure S3S, Supporting Information).

### 2.7 ZDHHC18 Palmitoylates MDH2 and Inhibits MDH2 Ubiquitination

**Figure 7:**
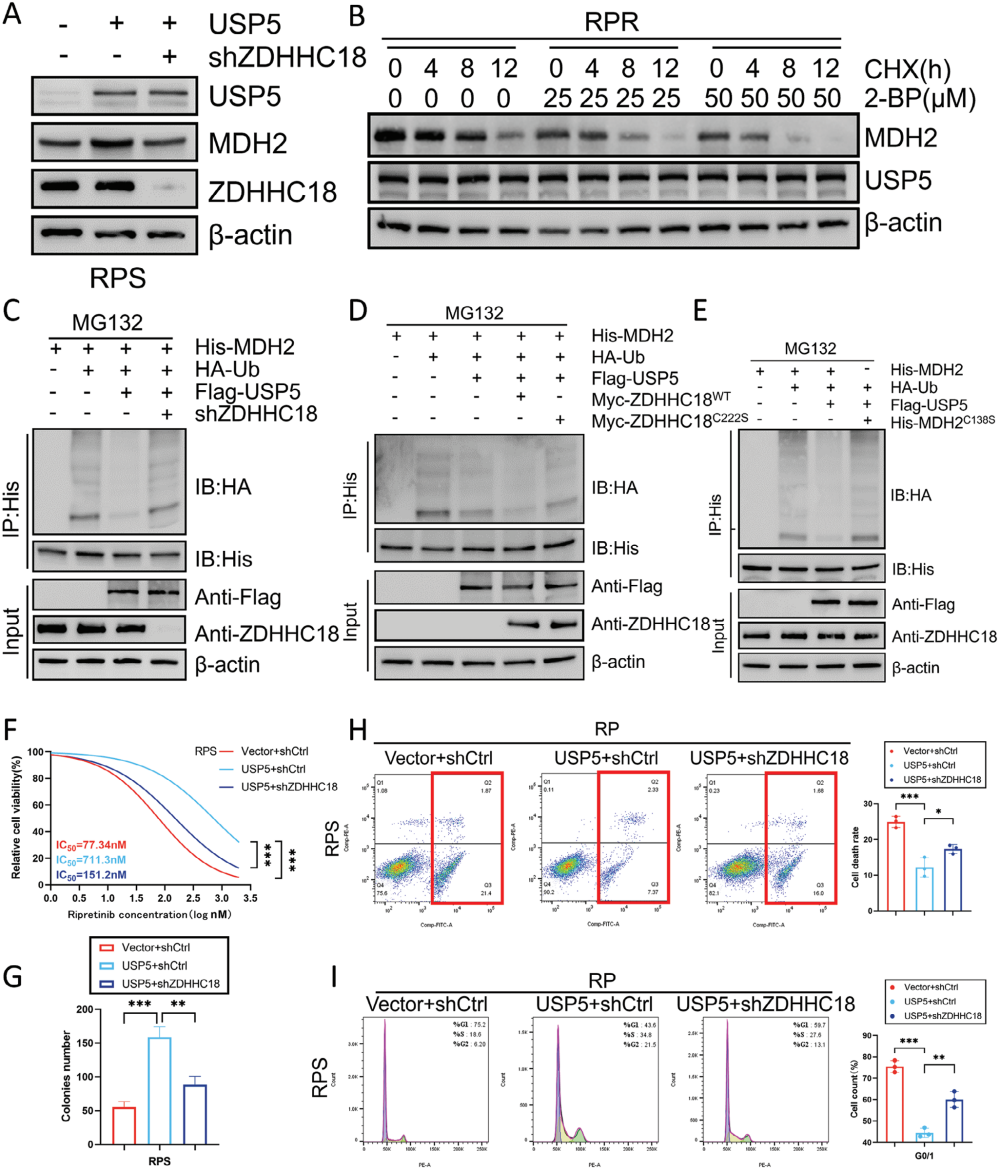
ZDHHC18 palmitoylates MDH2 and inhibits MDH2 ubiquitination A) The expression of MDH2 monitored by western blot. B) GIST‐RPR cells were treated with 2‐BP of the indicated concentration for 6 h. MDH2 protein level was determined by western blotting. C) GIST‐RPR cells were transfected with HA‐Ub and Flag‐USP5 plus control shRNA or ZDHHC18 shRNA. Then the cells were treated with MG132 for 8 h. Cell lysates were denature‐IP using an anti‐His antibody and then analyzed by western blotting using an anti‐HA antibody. D) GIST‐RPR cells were transfected with HA‐Ub, and wild‐type Myc‐ZDHHC18 or Myc‐ZDHHC18‐C222S, and then treated with MG132 for 8 h. Cell lysates were denature‐IP using an anti‐His antibody then analyzed by western blotting using an anti‐HA antibody. E) GIST‐RPR cells were transfected with HA‐Ub, wild‐type His‐MDH2 or His‐MDH2‐C138S, and then treated with MG132 for 8 h. Cell lysates were denature‐IP using an anti‐His antibody then analyzed by western blotting using an anti‐HA antibody. F) Cell viability of GIST‐RPS cells after treatment as indicated (mean ± SD, *n* = 3 independent experiments, one‐way ANOVA with Dunnett's multiple‐comparison test). ^***^ *p* < 0.001. G) The effect of ZDHHC18 expression levels on the proliferation of GIST cells was examined by clone formation assay (mean ± SD, *n* = 3 independent experiments, one‐way ANOVA with Dunnett's multiple‐comparison test). ^**^ *p* < 0.01, ^***^ *p* < 0.001. H,I) Cell cycle distribution and apoptosis rate were assessed in GIST‐RPS cells transduced with empty vector or USP5, and reconstituted with shCtrl or shZDHHC18 (mean ± SD, *n* = 3 independent experiments, one‐way ANOVA with Dunnett's multiple‐comparison test). ^*^ *p* < 0.05, ^**^P*p* < 0.01, ^***^ *p* < 0.001.

Several studies have revealed that palmitoylation might influence downstream protein degradation by affecting protein ubiquitination,^[^ ^22^ ^]^ and MDH2 can be palmitoylated by ZDHHC18.^[^ ^19^ ^]^ Thus, we hypothesized that ZDHHC18‐mediated palmitoylation at C138 influences the ubiquitination of MDH2 in GIST cells. In support of this hypothesis, overexpression of USP5 in sensitive cells led to an increase in MDH2 expression, whereas knocking down ZDHHC18 resulted in a decrease in MDH2 expression (**Figure** **7**A). Furthermore, in CHX‐treated resistant cells, the gradual addition of the palmitoylation inhibitor 2‐BP promoted MDH2 degradation (Figure 7B; Figure S3T, Supporting Information). The deubiquitination of MDH2 by USP5 was diminished following ZDHHC18 knockdown (Figure 7C). Moreover, the deubiquitination of MDH2 by USP5 was decreased following transduction with the catalytically inactive ZDHHC18 mutant (C222S) (Figure 7D). The C138S mutation in MDH2 similarly reduced the deubiquitination of MDH2 when ZDHHC18 was present (Figure 7E). These results confirm the critical role of ZDHHC18‐dependent palmitoylation of MDH2 in its deubiquitination by USP5.

Ultimately, our findings showed that the impact of USP5 overexpression on RP resistance was significantly counteracted by shZDHHC18 (Figures 7F–I, S3U). In summary, these findings underscore the crucial role of the USP5‐ZDHHC18‐MDH2 axis in driving malignant proliferation and fostering resistance to RP in GIST cells.

### 2.8 Correlations Among TRIM21, MDH2 and USP5 Expression and the Relationships of Their EXPRESSion with Progression‐Free Survival in Patients

**Figure 8:**
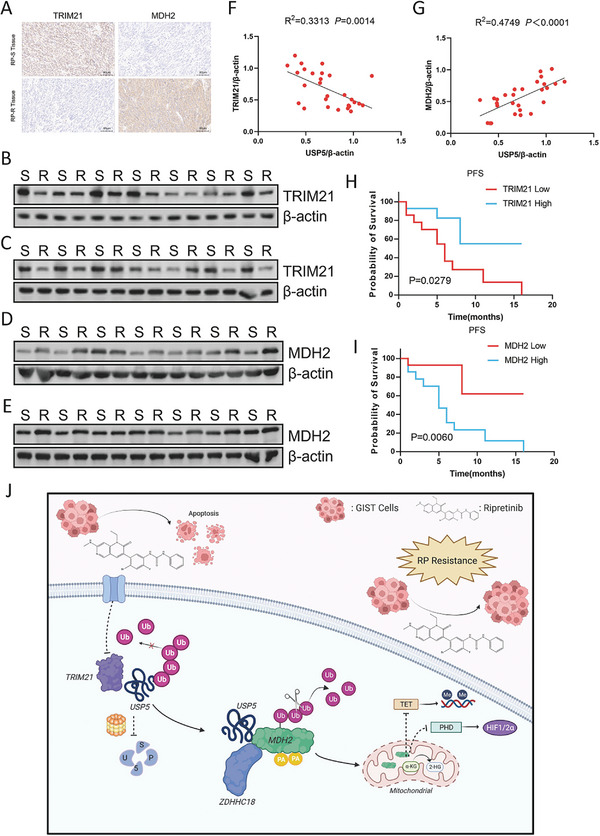
Correlation between TRIM21, MDH2, and USP5 expression and relationship with progression‐free survival of GISTs A) Representative images of IHC staining of TRIM21 and MDH2 in clinical GIST samples. Scale bars: 80 µm. B–E Protein levels of TRIM21 and MDH2 in human GIST tissues from RP‐resistant and RP‐sensitive patients. F) USP5 expression correlates negatively with TRIM21 expression in GIST patients (linear regression), *R* ^2^ = 0.3313, *p* = 0.0014. G) USP5 expression correlates positively with MDH2 expression in GIST patients (linear regression), *R* ^2^ = 0.4749, *p* < 0.0001. H) Kaplan–Meier plot of progression‐free survival by TRIM21 expression (Kaplan–Meier model with two‐sided log‐rank test). *p* = 0.0279. I) Kaplan–Meier plot of progression‐free survival by MDH2 expression (Kaplan–Meier model with two‐sided log‐rank test). *p* = 0.0060. J) The mechanistic scheme of this study.

Through IHC analysis of GIST tissues, we found low expression of TRIM21 and high expression of MDH2 in resistant tumor tissues (**Figure** **8**A). We utilized western blotting to measure the protein levels of TRIM21 and MDH2 in 14 pairs of GIST specimens to attempt to translate our findings into clinical applications. TRIM21 expression exhibited a negative correlation with USP5 expression, whereas MDH2 expression showed a positive correlation with USP5 expression (Figure 8B–G), revealing that patients with high TRIM21 expression commonly experienced extended progression‐free survival, while MDH2 expression was more strongly associated with poor prognosis (Figure 8H,I).

## 3 Discussion

In the Chinese population, the observed median progression‐free survival (mPFS) time of patients with GIST after treatment with ripretinib was 7.7 months, demonstrating good efficacy in this population as well.^[^ ^23^ ^]^ Additionally, studies have directly compared ripretinib with the second‐line drug sunitinib, demonstrating that the PFS of patients treated with ripretinib was not superior to that of patients treated with sunitinib, with a notable reduction in treatment‐emergent adverse events associated with ripretinib treatment.^[^ ^24^ ^]^ With the expanding application of ripretinib in patient populations, the issue of drug resistance is becoming increasingly important. Currently, research indicates that KIT remains the central oncogenic driver even in the later stages of GIST therapy.^[^ ^25^ ^]^ This study, unlike our study focusing on patients without secondary mutations, demonstrates that AP/AL mutations were rarely observed in progressing GIST samples from the pre‐ripretinib era, but in tumors from patients resistant to ripretinib, AP/AL mutations accounted for 50% of secondary KIT mutations. This finding provides a crucial theoretical basis for exploring ripretinib resistance in gastrointestinal stromal tumors and has significant clinical implications for guiding treatment strategies. Patients with secondary mutations have clearer resistance mechanisms, allowing for targeted drug use or combination therapy against the new mutation sites. Previously, USP5 was reported to exert oncogenic effects on various types of tumors, particularly breast cancer and non‐small cell lung cancer.^[^ ^26^ ^]^ Our research elucidated the role of USP5 in conferring resistance to ripretinib in GIST. On the one hand, USP5 is regulated by TRIM21, and on the other hand, it stabilizes the downstream protein MDH2, playing a crucial bridging role between these upstream and downstream components.

The unusually elevated protein level of MDH2 indicates its enzymatic promiscuity in catalyzing α‐ketoglutarate (α‐KG) to the oncometabolite 2‐hydroxyglutarate (2‐HG).^[^ ^21^ ^]^ We substantiated this inference in GIST cell lines. Interestingly, wild‐type GIST is a common resistant subtype of GIST that is resistant to first‐ to fourth‐line drugs, and α‐KG appears to be closely associated with the drug resistance mechanism in wild‐type GIST. Notably, among wild‐type GISTs, the SDH‐deficient subtype is the most common.^[^ ^27^ ^]^ Dysfunction of the SDH complex leads to intracellular accumulation of succinate, which inhibits various α‐KG‐dependent dioxygenases and their downstream enzymatic reactions within the cell.^[^ ^28^ ^]^ For instance, deficiency of the TET family of 5‐methylcytosine (5mC) hydroxylases induces high methylation levels of DNA and histones.^[^ ^29^ ^]^ Similarly, the lack of PHD (prolyl hydroxylases) leads to the accumulation of HIF1α.^[^ ^30^ ^]^ These are considered potential reasons for drug resistance in wild‐type GISTs.^[^ ^31^ ^]^ Our experimental findings suggest that RP‐resistant GIST cells exhibit heightened levels of MDH2, facilitating the conversion of α‐KG to 2‐HG. The reduction in α‐KG during this process may impact the activity of α‐ketoglutarate‐dependent dioxygenases, implicating the TRIM21–USP5–MDH2–α‐KG axis as a mechanism underlying cellular drug resistance. Moreover, the increased amount of 2‐HG may act as a competitive inhibitor of α‐KG to bind to α‐KG‐dependent dioxygenases and function as a competitive inhibitor of these enzymes,^[^ ^32^ ^]^ possibly further inhibiting enzyme activity and augmenting the resistance of cells to RP. Therefore, our findings suggest that treatment strategies for wild‐type GIST after the development of resistance to ripretinib are worth considering and may help in managing advanced ripretinib‐resistant GIST.

Palmitoylation, an increasingly explored lipid posttranslational modification, substantially affects protein localization, accumulation, secretion, and function. This process significantly impacts tumor occurrence and progression as well as therapeutic responses.^[^ ^33^ ^]^ Both palmitoylation and ubiquitination are posttranslational modifications (PTMs). Several studies currently indicate that palmitoylation can either promote or inhibit the ubiquitination of substrate proteins.^[^ ^22^ ^]^ After reviewing the literature, we discovered that MDH2 expression can be regulated by ZDHHC18‐mediated palmitoylation.^[^ ^19^ ^]^ Subsequently, we validated this conclusion, revealing that ZDHHC18‐mediated palmitoylation of MDH2 can synergize with its UPS5‐mediated deubiquitination in GIST cell lines.

In conclusion, this study identified ubiquitination as a key mechanism in ripretinib resistance in GIST. The deubiquitinase USP5 is regulated by TRIM21‐mediated ubiquitination while also deubiquitinating MDH2. This process stabilizes the MDH2 protein, promoting cell proliferation and drug resistance, while ZDHHC18‐mediated palmitoylation of MDH2 inhibits the ubiquitination of MDH2 (Figure 8J). Hence, targeting the USP5‐MDH2 axis could constitute a therapeutic strategy to address ripretinib resistance in GIST patients in the future.

## 4 Experimental Section

### Patients and Tissues

All tissue samples of gastrointestinal stromal tumors (GISTs) derived from 28 patients, namely, 14 ripretinib‐sensitive and 14 ripretinib‐resistant individuals, were procured from the First Affiliated Hospital of Nanjing Medical University, China. The baseline tumor sizes were established via CT prior to the initiation of ripretinib treatment. Regular CT scans were obtained during the course of ripretinib treatment for follow‐up assessments. Ripretinib‐sensitive patients were identified by either the stabilization or reduction of the tumor volume between successive CT scans, while resistant patients showed evident disease progression in their CT scans.

Tissue samples from patients in the sensitive group were obtained intraoperatively, whereas samples from patients in the resistant group were obtained during surgery or CT‐guided biopsy. Immediately after biopsy or resection, the tissues were preserved in liquid nitrogen. A comprehensive overview of the clinical features of the GIST patients is provided in Table S5 (Supporting Information). All patients included in this study were non‐secondary mutation patients, and there were no changes in their genotype before and after medication. Patient consent was obtained and the study was approved by the Ethics Committee of the First Affiliated Hospital of Nanjing Medical University prior to commencement.

### Primary Tumor Cell Culture

GIST tumor samples were acquired from patients undergoing surgical resection. Tumor tissues were preserved in serum‐free DMEM/F12 medium and fragmented into small pieces (5 mm^3^) using sterile scalpels or scissors. Collagenase type I was added to the tissues at concentrations ranging from 50 to 200 U mL^−1^ with 3 mmol L^−1^ CaCl_2_, and the tissues were incubated at 37 °C for 6 h. Cells were dispersed by passing the tissue lysate through a cell strainer and washed multiple times by centrifugation in PBS. The dispersed cells were then seeded into a culture dish containing DMEM/F12. Subsequently, the cells were cultured in DMEM/F12 supplemented with 10% FBS and 1% penicillin–streptomycin solution at 37 °C in a 5% CO_2_ atmosphere. Primary cell lines established from sensitive patients were designated as GIST‐RPS, whereas those derived from resistant patients were referred to as GIST‐RPR.

### Antibodies and Reagents

The antibodies used in study are shown in Table S6 (Supporting Information). The proteasome inhibitor MG132, cycloheximide (CHX), the protein synthesis inhibitor G9 (EOAI3402143), the USP5 inhibitor LW6 (CAY10585), and the palmitoylation inhibitor 2‐BP (2‐bromohexadecanoic acid) were purchased from Selleckchem (Houston, USA) and Cell Signaling Technology.

### Plasmids, Lentiviral Vectors, shRNAs and Transduction

Lentiviral expression vectors carrying Flag‐labeled TRIM21, Flag‐labeled TRIM21CA (C16A/C31A/H33W), His‐labeled USP5, Flag‐labeled USP5, Flag‐labeled USP5 W209A, and His‐labeled MDH2 were produced by cloning the respective genes with an N‐terminal Flag or His sequence into the pCDH‐CMV‐MCS‐EF1α‐Puro vector. The HA‐labeled ubiquitin‐Lys48 (K48) and HA‐labeled ubiquitin‐Lys63 (K63) constructs were acquired from Addgene (Watertown, USA). These constructs retained one wild‐type Lys residue (K48 or K63) while the remaining residues were substituted with Arg. All the constructs were validated by sequencing. Lentiviral plasmids carrying shRNA targeting USP5, TRIM21, or MDH2 or the nonspecific control shRNA were procured from Dharmacon (Shanghai, China). The shRNA sequences used are shown in Table S7 (Supporting Information). The transfection procedure involved the use of Lipo3000 (Invitrogen, Carlsbad, USA) following the manufacturer's protocol. LV‐Flag‐labeled USP5, LV‐Flag‐labeled USP5 W209A, LV‐Flag‐labeled TRIM21, LV‐Flag‐labeled TRIM21CA, LV‐His‐labeled USP5, and LV‐His‐labeled MDH2 were transfected into GIST‐RPS, GIST‐RPR, and HEK293T cells using a multiplicity of infection. An empty vector served as the transfection control. After screening with 2 µg mL^−1^ puromycin for 5–7 days, the transfected cells were collected. The TRIM21/USP5/MDH2 knockout GIST‐RPS and GIST‐RPR cell lines were screened using puromycin two days after lentivirus infection.

### RNA Isolation and Quantitative Real‐Time PCR

Total RNA was isolated from both cell lines and tissues using a Total RNA Purification Kit (Norgen, Thorold, Canada). Quantitative real‐time PCR (qRT‒PCR) was performed on a StepOnePlus Real‐Time PCR System (Thermo Fisher Scientific, Waltham, USA). The target mRNA levels were normalized to the GAPDH levels. The primers used for qPCR are shown in Table S8 (Supporting Information).

### IC_50_ and Resistance Index Calculations

The IC_50_ values and resistance indices of the GIST cells were assessed using a CCK‐8 assay (CK04, Dojindo, Tokyo, Japan). Briefly, GIST cells were plated into 96‐well plates in 100 µL of DMEM/F12 per well, followed by the addition of 10 µL of CCK‐8 reagent. After culture for 2 h, the assay was conducted. The optical density (OD450) of each well was measured using a microplate reader (Thermo Fisher Scientific, USA). Assays were performed in duplicate and repeated at least three times.

### Cell Cycle and Apoptosis Analyses

GIST cells were subjected to cell cycle analysis using a Cell Cycle Staining Kit (MultiSciences Biotech, Hangzhou, China) according to the manufacturer's instructions, followed by fluorescence‐activated cell sorting (FACS) analysis. For the apoptosis analysis, GIST cells were stained with Annexin V‐FITC and PI (Keygentec, Nanjing, China) as per the supplier's instructions. Subsequently, FACS analysis was conducted.

### Colony Formation Assay

A total of 1000 cells per well were plated in a six‐well plate and cultured in 2 mL of complete DMEM/F12. After the formation of visible colonies, the cells were fixed with 4% paraformaldehyde. After discarding the fixative solution, the samples were gently rinsed with tap water and then stained with crystal violet. Fourteen days later, images were acquired following drying at room temperature.

### Western Blot

Proteins were extracted from GIST cells and tissues by lysing the cells or tissues with RIPA lysis buffer (Beyotime, Nanjing, China) following the manufacturer's protocols. Proteins in the obtained lysates were separated by SDS‒PAGE and subjected to 80 V to facilitate migration. Subsequently, the separated proteins were transferred onto PVDF membranes (Millipore, Massachusetts, USA). The PVDF membranes were blocked with QuickBlock Blocking Buffer for 20 min and then incubated using primary antibodies at a temperature of 4 °C overnight. Following the washing steps (3 times for 15 min each) with TBST, the membranes were incubated with the specific secondary antibody (mouse or rabbit immunoglobulin). Super ECL Plus Kit (US EVERBRIGHT INC, Suzhou, China) was utilized to detect the protein bands on the membranes.

### Coimmunoprecipitation

Cells were collected and lysed for 35 min on ice using NP‐40 lysis buffer (Beyotime). Next, the lysates were centrifuged (12000 × g, 15 min), and the resulting supernatant was incubated with a specific antibody with rotation (4 °C, 10 h). Protein A/G Plus Agarose (Thermo Fisher Scientific) was added, and the mixture was incubated at 4 °C overnight with rotation. Subsequently, the Protein A/G Plus Agarose mixture was thoroughly washed with 1 mL wash buffer three times (4 °C, 20 min each). After removal of the wash buffer, the samples were treated with 2X SDS‒PAGE Sample Buffer (Beyotime) and incubated (95 °C, 9 min).

### Denaturing IP Assay

All ubiquitination assays in our investigation were conducted under denaturing conditions utilizing d‐IP for detection. Cells were lysed in SDS‐denaturing buffer (containing 62.5 mm Tris‐HCl (pH 6.8), 2% SDS, 10% glycerol, and 1.5% β‐mercaptoethanol) and boiled for 10 min. Following this step, the cell lysates were diluted tenfold to fortyfold in native lysis buffer (composed of 50 mm Tris‐HCl (pH 7.4), 0.5% Triton X‐100, 200 mm NaCl, and 10% glycerol). Subsequently, the supernatants obtained after centrifugation at 13000 revolutions per minute for 5 min were subjected to immunoprecipitation. The lysates and immune complexes were analyzed using western blotting.

### Protein Half‐Life Assay

For half‐life assays, the specified GIST cells were treated with CHX (40 µg/ml) for the specified durations prior to collecting cell lysates.

### Immunohistochemistry

Fresh‐frozen human GIST tissue samples were fixed with 4% formalin and embedded in paraffin for immunohistochemical (IHC) staining. Four‐micron‐thick sections sliced from paraffin‐embedded tissues were subjected to staining with specific primary antibodies overnight at 4 °C. After the overnight incubation, the sections were incubated with HRP‐conjugated secondary antibodies at 37 °C for 1 h in the dark. Subsequently, a counterstain with Hoechst nuclear stain (Thermo Fisher Scientific, MA, USA) was applied to the tissues for 1 min. Images of the sections were acquired using a fluorescence microscope at a magnification of 40x.

### Mitochondrial Malate Dehydrogenase Activity Assay

The impact of LW6 on MDH2 activity was assessed using cell extracts cultured in 6‐well plates under normoxic conditions with various concentrations of LW6. A MDH2 assay kit (ab119693, Abcam) was used to measure MDH2 activity. These experiments were repeated three times to confirm accuracy and reliability.

### Subcutaneous Xenograft Models

All research involving animals adhered to ethical standards and was approved by the Nanjing Medical University Institutional Animal Care and Use Committee. To establish the subcutaneous xenograft models, 6‐week‐old female nude mice were injected subcutaneously with GIST cells in the logarithmic growth phase (5 × 10^6^ cells per mouse) suspended in 100 µL of phosphate‐buffered saline (PBS) for tumor growth studies. Treatment commenced once the tumor volume reached ≈200 mm^3^. The mice were monitored daily to observe subcutaneous tumor development. After the tumors reached the specified size, the mice were euthanized. The mice were treated with ripretinib at a dosage of 100 mg kg^−1^/day once the tumors reached the desired size. Additionally, the USP5 inhibitor G9 was administered at a dosage of 15 mg kg^−1^/day, and the MDH2 inhibitor LW6 was administered at a dosage of 20 mg kg^−1^/day as needed.

### Statistical Analysis

The data are presented as the mean ± SD from at least three independent experiments. Statistical analyses were carried out using GraphPad Prism 9.2 and SPSS 26.0 software, employing two‐tailed Student's *t*‐test or one‐way ANOVA. Specifically, the statistical analysis involved the use of a two‐tailed unpaired Student's t‐test to compare two groups of data, and a one‐way ANOVA with Dunnett's or Tukey's multiple‐comparison test was used for comparing multiple groups of data. A *p*‐value of <0.05 indicated a statistically significant difference.

### Ethics approval and Consent to Participate

The collection of specimens and animal handling for the study were reviewed and approved by the Ethics Committee of the First Affiliated Hospital of Nanjing Medical University.

## Conflict of Interest

The authors declare no conflict of interest.

## Author Contributions

H.S., Z.C., C.L., Z.G., and J.X. contributed equally to this work. H.Y.S. conceptualized and designed the project; H.Y.S., Z.W.C., J.X., H.X., B.W.L., and F.Y.L. performed data acquisition, and interpretation and analyzed the project; H.Y.S., C.L., Z.S.G., J.X., Y.B.B., J.N.Z, T.Y.L., Q.Z.Z., and Z.Y.H. performed investigation; C.L., Z.S.G., T.H.G., D.H.Y., Z.K.X., and H.X. performed acquisition of patient specimens; H.Y.S., Z.W.C., C.L., Z.S.G., and J.X. did article drafting and revised the project; HYS wrote the article. All authors approved the final version of the manuscript.

## Supporting information

Supporting Information

## Data Availability

The data that support the findings of this study are available from the corresponding author upon reasonable request.
