# Supplementary material for: USP5 Promotes Ripretinib Resistance in Gastrointestinal Stromal Tumors by MDH2 Deubiquition
Source: Adv Sci (Weinh). 2024 Jul 8;11(34):2401171. doi: 10.1002/advs.202401171 (PMC11425886; doi:10.1002/advs.202401171)
Supplement: Supplementary file 1 — Supporting Information [file ADVS-11-2401171-s001.docx]

**Figure S1**


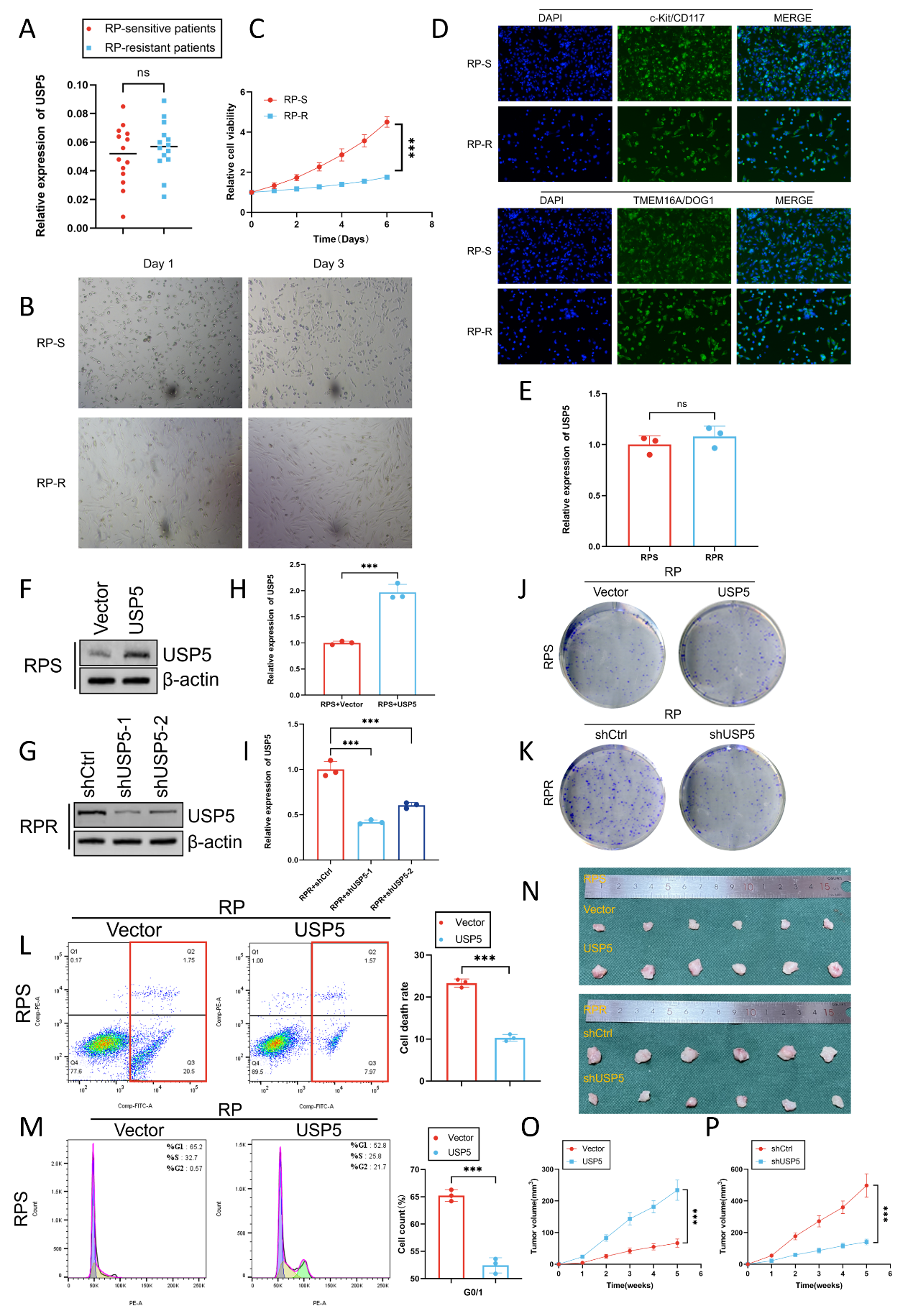


**Figure S2**


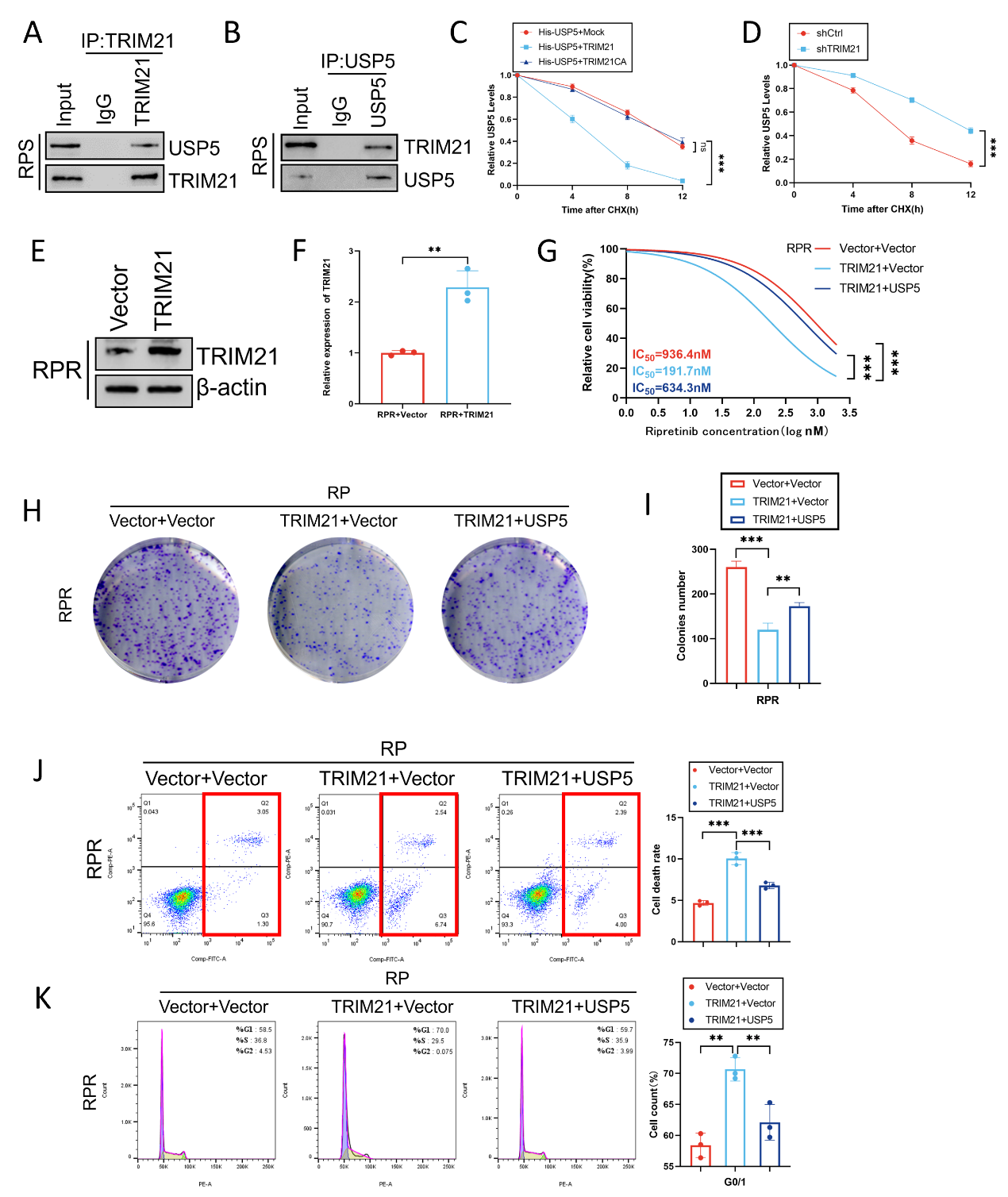


**Figure S3**


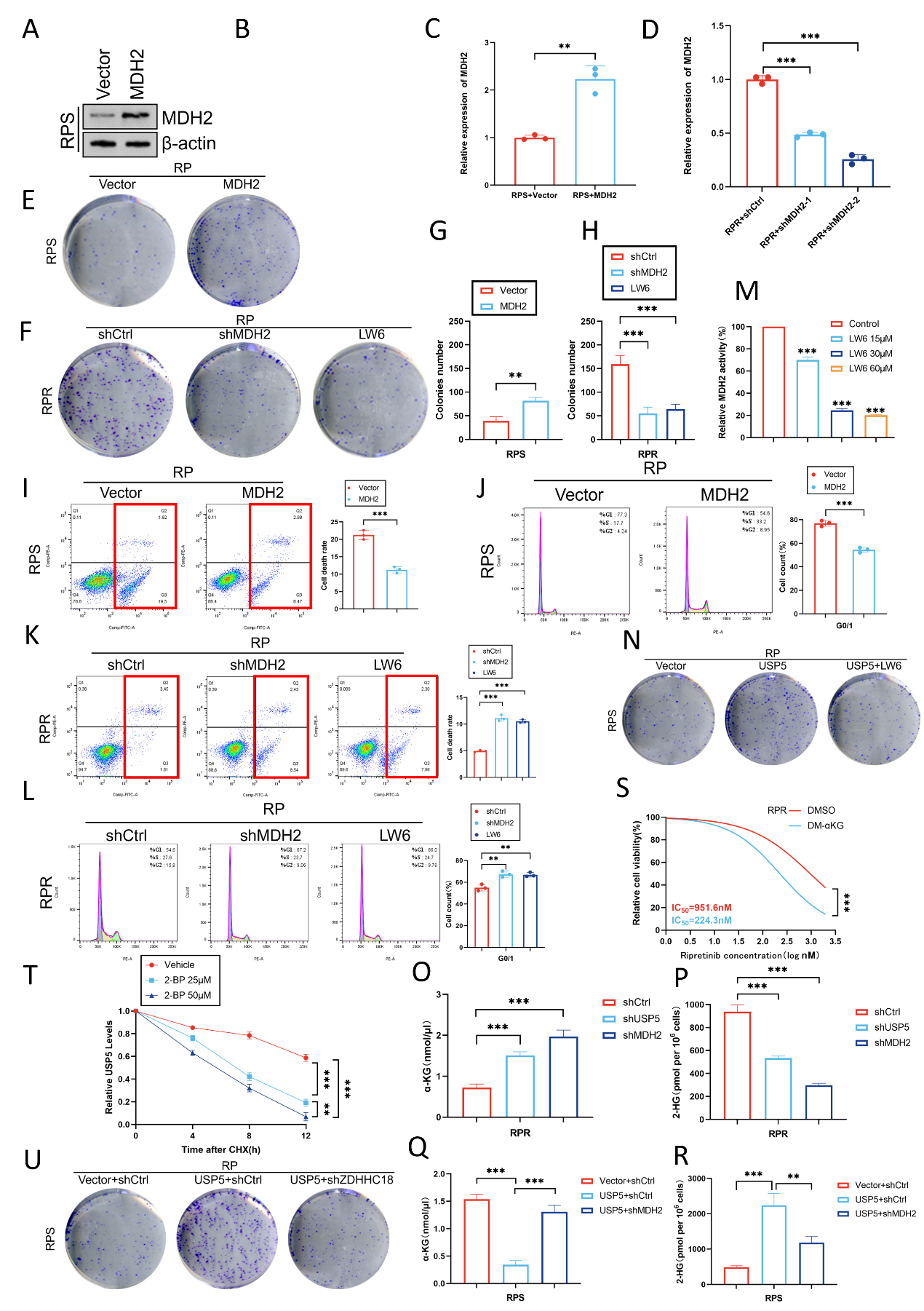


**Fig. S1 A** RT-qPCR analysis of USP5 mRNA levels in sensitive and resistant GIST tissues (mean ± SD, n = 14 patients for each group, two-tailed Student’s t-test). ns: P ≥ 0.05. **B** Morphology of primary GIST cells under a 40x magnification. **C** Cell viability assay of primary GIST cells (mean ± SD, n = 3 independent experiments, two-tailed Student’s t-test). ***P < 0.001. **D** Immunofluorescence targeting DOG1 and CD117 in primary GIST cells under a 100x magnification. **E** RT-qPCR analysis of USP5 target genes from RP-sensitive and RP-resistant cells (mean ± SD, n = 3 independent experiments, two-tailed Student’s t-test). ns: P ≥ 0.05. **F, G** Protein level of USP5 overexpressing and knockdown efficiency in GIST cells. **H** RT-qPCR analysis of USP5 overexpressing efficiency in GIST cells (mean ± SD, n = 3 independent experiments, two-tailed Student’s t-test). ***P < 0.001. **I** RT-qPCR analysis of USP5 knockdown efficiency in GIST cells (mean ± SD, n = 3 independent experiments, one-way ANOVA with Dunnett’s multiple-comparison test). ***P < 0.001. **J, K** Clone formation verified the effect of USP5 expression levels on the proliferation of GIST cells after treatment with ripretinib (80 nM) for 24 h. **L, M** Apoptosis rate and cell cycle distribution of RP-sensitive GIST cells transfected with vector or USP5 after treatment with ripretinib (80 nM) for 24 h (mean ± SD, n = 3 independent experiments, two-tailed Student’s t-test). ***P < 0.001. **N** Representative images of tumors in nude mice bearing GIST cells in different groups (n = 6 mice/group). Scale bars: 1 cm. **O, P** Average volume of the excised tumors for each group (mean ± SD, n = 6 mice for each group, two-tailed Student’s t-test). ***P < 0.001.

**Fig. S2 A, B** Cell lysates from GIST-RPS cells were analyzed by IP using antibodies against TRIM21 and USP5, then subjected to western blotting analysis. IgG was used as the isotype control. **C** HEK293T cells were co-transfected with His-labeled USP5 and Flag-labeled wild-type TRIM21 or TRIM21CA, treated with CHX (40 μg/ml), collected at the indicated times, and then subjected to western blotting with antibodies against His and Flag. Quantification of USP5 levels relative to β-actin are shown (mean ± SD, n = 3 independent experiments, one-way ANOVA with Dunnett’s multiple-comparison test). ***P < 0.001 ns: P ≥ 0.05. **D** GIST-RPS cells stably expressing control shRNA or shRNA-TRIM21 were treated with CHX (40 μg/ml), harvested at the indicated times, and then subjected to western blotting with antibodies against USP5 and TRIM21. Quantification of USP5 levels relative to β-actin are shown (mean ± SD, n = 3 independent experiments, two-tailed Student’s t-test). ***P < 0.001. **E** Protein level of TRIM21 overexpressing efficiency in GIST-RPR. **F** RT-qPCR analysis of TRIM21 overexpressing efficiency in GIST-RPR (mean ± SD, n = 3 independent experiments, two-tailed Student’s t-test). **P < 0.01. G Cell viability of GIST cells after treatment as indicated (mean ± SD, n = 3 independent experiments, one-way ANOVA with Dunnett’s multiple-comparison test). ***P < 0.001. **H, I** Clone formation assessed in GIST-RPR cells transduced with empty vector, TRIM21 or USP5 after treatment with ripretinib (80 nM) for 24 h (mean ± SD, n = 3 independent experiments, one-way ANOVA with Dunnett’s multiple-comparison test). **P < 0.01, ***P < 0.001. **J, K** Apoptosis rate and cell cycle distribution assessed in GIST-RPR cells transduced with empty vector, TRIM21 or USP5 after treatment with ripretinib (80 nM) for 24 h (mean ± SD, n = 3 independent experiments, one-way ANOVA with Dunnett’s multiple-comparison test). **P < 0.01, ***P < 0.001.

**Fig. S3 A, B** Protein level of MDH2 overexpressing and knockdown efficiency in GIST cells. **C** RT-qPCR analysis of MDH2 overexpressing efficiency in GIST cells (mean ± SD, n = 3 independent experiments, two-tailed Student’s t-test). **P < 0.01. **D** RT-qPCR analysis of MDH2 knockdown efficiency in GIST cells (mean ± SD, n = 3 independent experiments, one-way ANOVA with Dunnett’s multiple-comparison test). ***P < 0.001. **E, G, I, J** Clone formation, cell cycle distribution and apoptosis rate were assessed in GIST cells transduced with empty vector or MDH2 after treatment with ripretinib (80 nM) for 24 h (mean ± SD, n = 3 independent experiments, two-tailed Student’s t-test). **P < 0.01, ***P < 0.001. **F, H, K, L** Clone formation, cell cycle distribution and apoptosis rate were assessed in GIST cells reconstituted with shCtrl or shMDH2 or treated with LW6 (30 µM) after treatment with ripretinib (80 nM) for 24 h (mean ± SD, n = 3 independent experiments, one-way ANOVA with Dunnett’s multiple-comparison test). **P < 0.01, ***P < 0.001. **M** MDH2 activity was assessed in cell lysates from GIST-RPR cultured under normoxia with or without LW6 at a concentration of 15, 30, or 60 µM (mean ± SD, n = 3 independent experiments, one-way ANOVA with Dunnett’s multiple-comparison test). ***P < 0.001. **N** Clone formation was assessed in GIST-RPS cells transduced with empty vector or USP5 with or without LW6 (30 µM). **O-R** Intracellular α-KG and 2-HG level in GIST cells transfected with shCtrl, shUSP5, shMDH2, vector or USP5 (mean ± SD, n = 3 independent experiments, one-way ANOVA with Dunnett’s multiple-comparison test). **P < 0.01, ***P < 0.001. **S** CCK8 proliferation assay was accessed in GIST-RPR cells with or without with 4 mM DM-αKG for 72 h (mean ± SD, n = 3 independent experiments, two-tailed Student’s t-test). ***P < 0.001. **T** MDH2 protein level was determined by western blotting. Quantification of MDH2 levels relative to β-actin are shown (mean ± SD, n = 3 independent experiments, one-way ANOVA with Tukey’s multiple-comparison test). **P < 0.01, ***P < 0.001. **U** The effect of ZDHHC18 expression levels on the proliferation of GIST cells was examined by clone formation assay.

**Table S1**

Genetic sequencing of primary cell lines

| Id | Type | Gene | Gene.ID | AAChange |
| --- | --- | --- | --- | --- |
| RP-R | Mutant | KIT | KIT:NM_000222.2:exon13 | p.V654A |
| RP-R | Mutant | KIT | KIT:NM_000222.2:exon11 | p.Q556_K558delinsP |
| RP-S | Mutant | KIT | KIT:NM_000222.2:exon13 | p.K642E |

**Table S2**

The mass spectrometry results of USP5

| Accession | Description | Abundances |
| --- | --- | --- |
| P45974 | Ubiquitin carboxyl-terminal hydrolase 5 | 1563416028.5 |
| P19474 | E3 ubiquitin-protein ligase TRIM21 | 15971208 |

**Table S3**

Predictions of USP5 mutants from GPS-Uber

| Id | Position | Code | Peptide | Score |
| --- | --- | --- | --- | --- |
| USP5_  HUMAN | 743 | K | SMGFSRDQALKALRATNNSLE | 0.7115 |
| USP5_  HUMAN | 423 | K | APRMFKALIGKGHPEFSTNRQ | 0.6836 |
| USP5_  HUMAN | 406 | K | SGDGERVPEQKEVQDGIAPRM | 0.6759 |

**Table S4**

Predictions of MDH2 mutants from GPS-Uber

| Id | Position | Code | Peptide | Score |
| --- | --- | --- | --- | --- |
| MDH2_  HUMAN | 185 | K | VRANTFVAELKGLDPARVNVP | 0.6784 |
| MDH2_  HUMAN | 239 | K | RIQEAGTEVVKAKAGAGSATL | 0.5269 |
| MDH2_  HUMAN | 335 | K | ASIKKGEDFVKTLK | 0.4006 |

**Table S5**

Clinical features of the GIST patients

| Patient | Date of diagnosis | Date of disease progression | Progression-free survival period |
| --- | --- | --- | --- |
| 1 | 2021/7/21 | 2022/1/19 | 5 |
| 2 | 2022/1/22 | 2022/8/5 | 6 |
| 3 | 2021/11/28 | 2021/12/30 | 1 |
| 4 | 2022/2/1 | 2022/5/23 | 3 |
| 5 | 2021/6/9 | 2021/11/16 | 5 |
| 6 | 2021/12/20 | 2022/2/12 | 1 |
| 7 | 2022/6/8 | 2022/8/7 | 1 |
| 8 | 2021/7/16 | 2021/9/19 | 2 |
| 9 | 2021/5/1 | 2022/4/7 | 11 |
| 10 | 2022/2/2 | 2022/10/17 | 8 |
| 11 | 2021/6/15 | 2022/11/6 | 16 |
| 12 | 2021/7/26 | 2022/1/9 | 5 |
| 13 | 2021/8/25 | 2022/3/27 | 7 |
| 14 | 2021/6/13 | 2022/1/9 | 6 |
| 15 | 2022/7/1 | No disease progression | None |
| 16 | 2021/9/19 | No disease progression | None |
| 17 | 2022/3/2 | No disease progression | None |
| 18 | 2022/3/13 | No disease progression | None |
| 19 | 2021/12/18 | No disease progression | None |
| 20 | 2021/9/7 | No disease progression | None |
| 21 | 2021/6/30 | No disease progression | None |
| 22 | 2021/12/13 | No disease progression | None |
| 23 | 2021/11/8 | No disease progression | None |
| 24 | 2022/1/18 | No disease progression | None |
| 25 | 2022/4/7 | No disease progression | None |
| 26 | 2021/6/18 | No disease progression | None |
| 27 | 2021/7/4 | No disease progression | None |
| 28 | 2021/8/18 | No disease progression | None |

**Table S6**

Antibodies used in our study

| Antibody | Type | Supplier | Product code |
| --- | --- | --- | --- |
| anti-USP5 | rabbit monoclonal | Abcam, Cambridge, UK | ab154170 |
| anti-TRIM21 | rabbit monoclonal | Abcam, Cambridge, UK | ab207728 |
| anti-MDH2 | rabbit monoclonal | Abcam, Cambridge, UK | ab181873 |
| anti-β-actin | rabbit polyclonal | Proteintech, Wuhan, China | 66009-1-Ig |
| anti-ZDHHC18 | rabbit polyclonal | Immunoway, Suzhou, China | YN1026 |
| anti-Myc | rabbit monoclonal | Abcam, Cambridge, UK | ab32072 |
| anti-His | mouse monoclonal | Abcam, Cambridge, UK | ab18184 |
| anti-GST | rabbit monoclonal | Abcam, Cambridge, UK | ab138491 |
| anti-Flag | rabbit monoclonal | Cell Signaling Technology | 14793 |
| anti-HA | rabbit monoclonal | Abcam, Cambridge, UK | ab236632 |
| anti-Ubiquitin | mouse monoclonal | Biolegend, San Diego, USA | 646302 |

**Table S7**

| shUSP5-1 | 5′-GATAGACATGAACCAGCGGAT-3′ |
| --- | --- |
| shUSP5-2 | 5′-GACCACACGATTTGCCTCATT-3′ |
| shTRIM21-1 | 5′-UCAUUGUCAAGCGUGCUGC-3′ |
| shTRIM21-2 | 5′-UGGCAUGGAGGCACCUGAAGGUGG-3′ |
| shMDH2-1 | 5′-GCCCAGAACAATGCTAAAGTA-3′ |
| shMDH2-2 | 5′-GAAGCCATGATCTGCGTCATT-3′ |
| shZDHHC18 | 5′-GTTTATTCTCTCCCTCTCATT-3′ |

ShRNA sequences used in our study

**Table S8**

| USP5 | Forward | 5′-GCTGCTGTCAGTATTACCGAC-3′ |
| --- | --- | --- |
|  | Reverse | 5′-AAAGCCCAGAAACGTGTTCATA-3′ |
| TRIM21 | Forward | 5′-CTTGCTTCTGAGCGGAAACT-3′ |
|  | Reverse | 5′-AGGCAGATAGGGCATGTGAC-3′ |
| MDH2 | Forward | 5′-CCCACGGGTTCATAGTTCAG-3′ |
|  | Reverse | 5′-CATCAGGGTTCGGTCAGAAG′ |
| GAPDH | Forward | 5′-GAGTCAACGGATTTGGTCGT-3′ |
|  | Reverse | 5′-GACAAGCTTCCCGTTCTCAG-3′ |

Primers used for qPCR
